# Kidney dysfunction under cardiometabolic stress promotes calorie-intake-independent wasting and frailty-like muscle phenotypes

**DOI:** 10.1016/j.isci.2026.118058

**Published:** 2026-10-30

**Authors:** Mitsuharu Kojima, Yuka Sato, Akiyoshi Hirayama, Akihiro Ryuge, Takahiro Imaizumi, Kayaho Maeda, Noritoshi Kato, Yohei Kozaki, Masahiro Sugimura, Yuhei Noda, Katsuaki Shibata, Chikao Onogi, Kota Tanabe, Mikito Takefuji, Kenya Terabe, Kazuhiro Furuhashi, Tomoki Kosugi, Naotake Tsuboi, Kenji Kadomatsu, Shoichi Maruyama

**Affiliations:** 1 Department of Nephrology, Nagoya University Graduate School of Medicine, Nagoya, Aichi 466-8550, Japan; 2 Department of Nephrology, School of Medicine, Fujita Health University, Toyoake, Aichi 470-1192, Japan; 3 Institute for Advanced Biosciences, Keio University, Tsuruoka, Yamagata 997-0052, Japan; 4 Department of Clinical Research Education, Nagoya University Graduate School of Medicine, Nagoya, Aichi 466-8550, Japan; 5 Department of Cardiology, Nagoya University Graduate School of Medicine, Nagoya, Aichi 466-8550, Japan; 6 Department of Orthopedic Surgery, Nagoya University Graduate School of Medicine, Nagoya, Aichi 466-8550, Japan; 7 Institute for Glyco-core Research (iGCORE), Nagoya University/Gifu University, Nagoya, Aichi 464-8601, Japan

**Keywords:** aging, chronic kidney disease, CKD, sarcopenia, frailty, glucose homeostasis, energy homeostasis, CKM, metabolic inflexibility, gluconeogenesis

## Abstract

Although age-related sarcopenia and frailty are linked to impaired metabolic flexibility and disrupted energy homeostasis, whether this framework explains chronic kidney disease (CKD)-related wasting remains unclear. We show that superimposed nephron loss redirects cardiometabolic stress from obesity and glucose intolerance toward calorie-intake-independent wasting with sarcopenic and physical frailty features. This switch was accompanied by impaired skeletal muscle glucose-utilization programs, a blunted glycemic response to gluconeogenic substrates, and altered hepatic-renal gluconeogenic programs. Integrated omics revealed suppressed glucose programs, impaired fatty acid utilization-related compensation, and amino acid catabolic signatures in skeletal muscle. Similar features occurred with aging plus nephron loss and in older human CKD kidneys with reduced renal PCK1 expression. Cornstarch-based nutritional modulation improved exercise capacity, muscle mass, and serum albumin, highlighting slow-release carbohydrate modulation as a potential therapeutic entry point. These findings support a model in which kidney dysfunction promotes wasting by limiting systemic metabolic reserve and skeletal muscle metabolic flexibility.

## Highlights

• Nephron loss shifts cardiometabolic stress toward calorie-intake-independent wasting
• Altered hepatic-renal glycemic programs accompany suppressed muscle glucose programs
• Similar wasting occurs when nephron loss is superimposed on aging
• Cornstarch-based nutrition partially restores muscle mass and physical performance

## Graphical abstract

**Figure:**
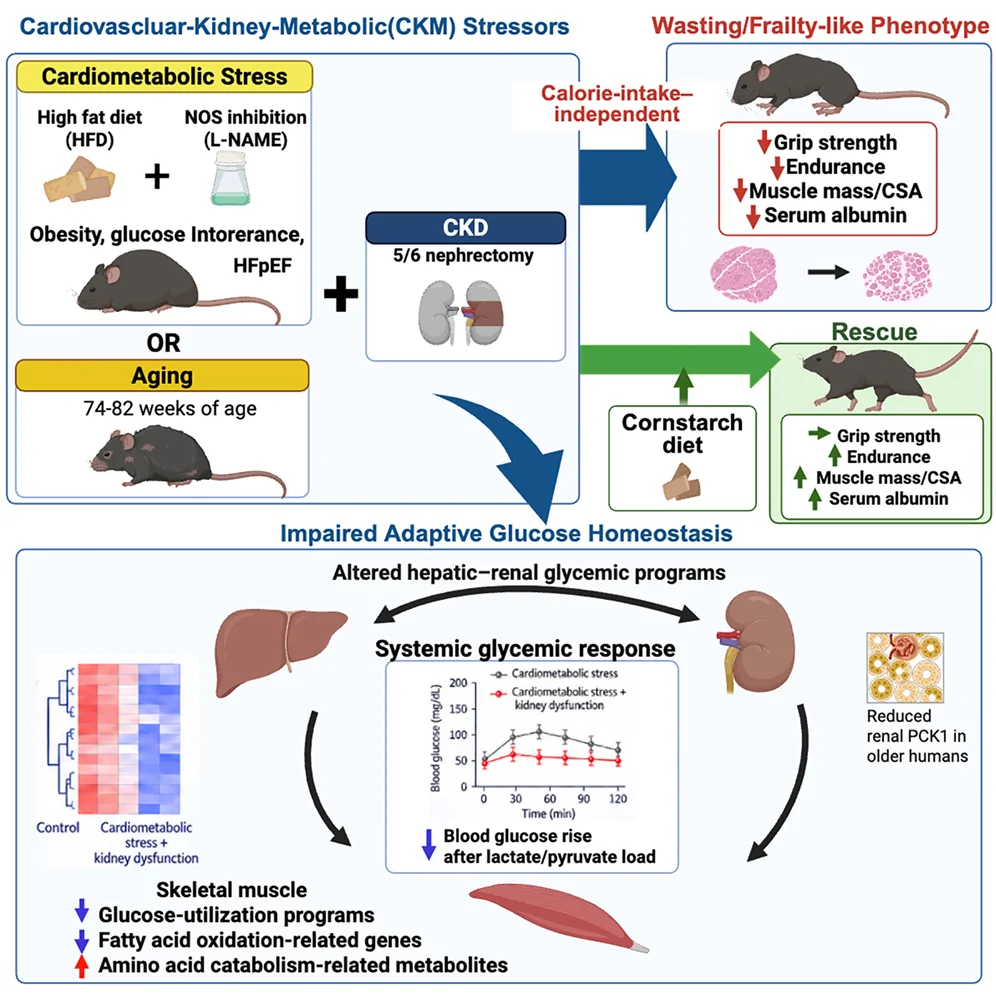

## Teaser

Health sciences; Endocrinology; Human metabolism

## Introduction

Metabolic flexibility, defined as the capacity to adapt fuel utilization to changing nutrient availability and energetic demand, is increasingly recognized as a key feature of metabolic health and aging-related disease.^1^^,^^2^^,^^3^ Loss of metabolic flexibility has been implicated in impaired energy homeostasis, reduced stress tolerance, and age-associated decline in skeletal muscle function.^4^ Sarcopenia and frailty may therefore reflect not only local muscle dysfunction, but also impaired systemic metabolic adaptation.^5^^,^^6^^,^^7^

Chronic kidney disease (CKD) is highly prevalent in older adults and is closely associated with sarcopenia, frailty, disability, and mortality.^8^^,^^9^^,^^10^^,^^11^ Although muscle wasting in CKD has traditionally been attributed to reduced physical activity, inflammation, metabolic acidosis, uremic toxins, malnutrition, and endocrine abnormalities,^12^^,^^13^^,^^14^^,^^15^ the mechanisms by which kidney dysfunction promotes progressive loss of muscle mass and function remain incompletely understood. In particular, whether CKD alters systemic energy metabolism and inter-organ fuel adaptation in a manner that predisposes to sarcopenia and physical frailty remains poorly defined.^16^

Understanding CKD-associated changes in systemic energy metabolism is challenging because CKD rarely occurs as an isolated metabolic condition. In clinical populations, CKD commonly develops in the context of aging and coexists with hypertension, vascular dysfunction, obesity, diabetes, dyslipidemia, and heart failure, a disease clustering increasingly conceptualized within the cardiovascular-kidney-metabolic (CKM) framework.^17^^,^^18^^,^^19^ These overlapping conditions can constrain systemic metabolic flexibility, inter-organ metabolic adaptation, and muscle homeostasis,^3^^,^^20^ yet how kidney dysfunction modifies tissue maintenance in this setting remains poorly understood. Epidemiological studies have consistently linked CKD to sarcopenia and frailty,^10^^,^^21^^,^^22^ but these associations are often observed in older individuals with substantial cardiometabolic comorbidity.^11^^,^^23^^,^^24^ Conversely, experimental nephron loss alone, including 5/6 nephrectomy, does not always reproduce overt sarcopenic phenotypes.^25^^,^^26^ These observations suggest that CKD may act as a permissive or amplifying factor, with overt wasting becoming manifest primarily when kidney dysfunction is superimposed on aging-related vulnerability or additional cardiometabolic stress.

The convergence of aging, cardiometabolic comorbidity, and nephron loss suggests that CKD-associated sarcopenia may emerge, at least in part, from impaired systemic fuel adaptation rather than from isolated muscle pathology alone. A central component of systemic fuel adaptation is the liver-muscle substrate cycle: skeletal muscle consumes glucose and supplies gluconeogenic substrates such as lactate, alanine, and glutamine during fasting or stress, whereas the liver uses these substrates to maintain systemic glucose availability.^27^ The kidney also contributes to endogenous glucose production, yet whether loss of renal metabolic reserve disrupts hepatic-renal-muscle fuel adaptation and contributes to CKD-associated wasting remains underexplored.^28^^,^^29^^,^^30^^,^^31^^,^^32^^,^^33^ Nephron loss may reduce renal metabolic reserve,^34^^,^^35^^,^^36^^,^^37^ while cardiometabolic stress can disrupt hepatic glucose and lipid metabolism.^38^ How hepatic and renal glucose-related metabolic responses interact with skeletal muscle fuel utilization and amino acid metabolism in CKD-associated wasting remains poorly defined.

Based on this framework, we investigated whether kidney dysfunction under cardiometabolic stress disrupts glucose-related inter-organ metabolic adaptation and promotes tissue wasting despite preserved caloric intake. To model this interaction, we combined 5/6 nephrectomy with high-fat diet and L-NAME treatment, an experimental approach that combines lipid overload with nitric oxide synthase inhibition-mediated vascular stress and recapitulates features of HFpEF cardiometabolic dysfunction.^39^ HFD + L-NAME treatment was used not to model aging itself, but to isolate and intensify a cardiometabolic-stress component frequently embedded within aging-associated CKD. We then examined aging-plus-nephron-loss mice and renal PCK1 expression in human kidney biopsy specimens to determine whether related nephron loss-associated metabolic vulnerability was also observed in physiological aging. Integrated metabolomic and transcriptomic profiling and cornstarch-based nutritional modulation were further used to define metabolic programs associated with this phenotype and their nutritional modifiability.

We show that nephron loss can shift the metabolic consequences of cardiometabolic stress from glucose intolerance and weight gain toward calorie-intake-independent wasting with sarcopenic and physical frailty features. This phenotypic shift was accompanied by skeletal muscle metabolic maladaptation and impaired adaptive glucose homeostasis, consistent with disrupted glucose-related inter-organ metabolic adaptation. Collectively, these findings support a working model in which superimposed kidney dysfunction limits systemic metabolic reserve and skeletal muscle metabolic flexibility, thereby contributing to tissue wasting despite preserved caloric intake.

## Results

### Kidney dysfunction under cardiometabolic stress is associated with calorie-intake-independent wasting and frailty-like muscle phenotypes

To model kidney dysfunction under cardiometabolic stress, we combined nephron loss with a high-fat diet and nitric oxide synthase inhibition (HFD + L-NAME). Four experimental groups were analyzed: control (C), nephrectomy (N), HFD + L-NAME (HL), and HFD + L-NAME with nephrectomy (HLN) (Figure 1A). Blood urea nitrogen and serum creatinine levels were comparably increased in N and HLN mice, indicating that HFD + L-NAME did not further exacerbate renal dysfunction beyond nephron loss itself (Figure 1B).

**Figure 1:**
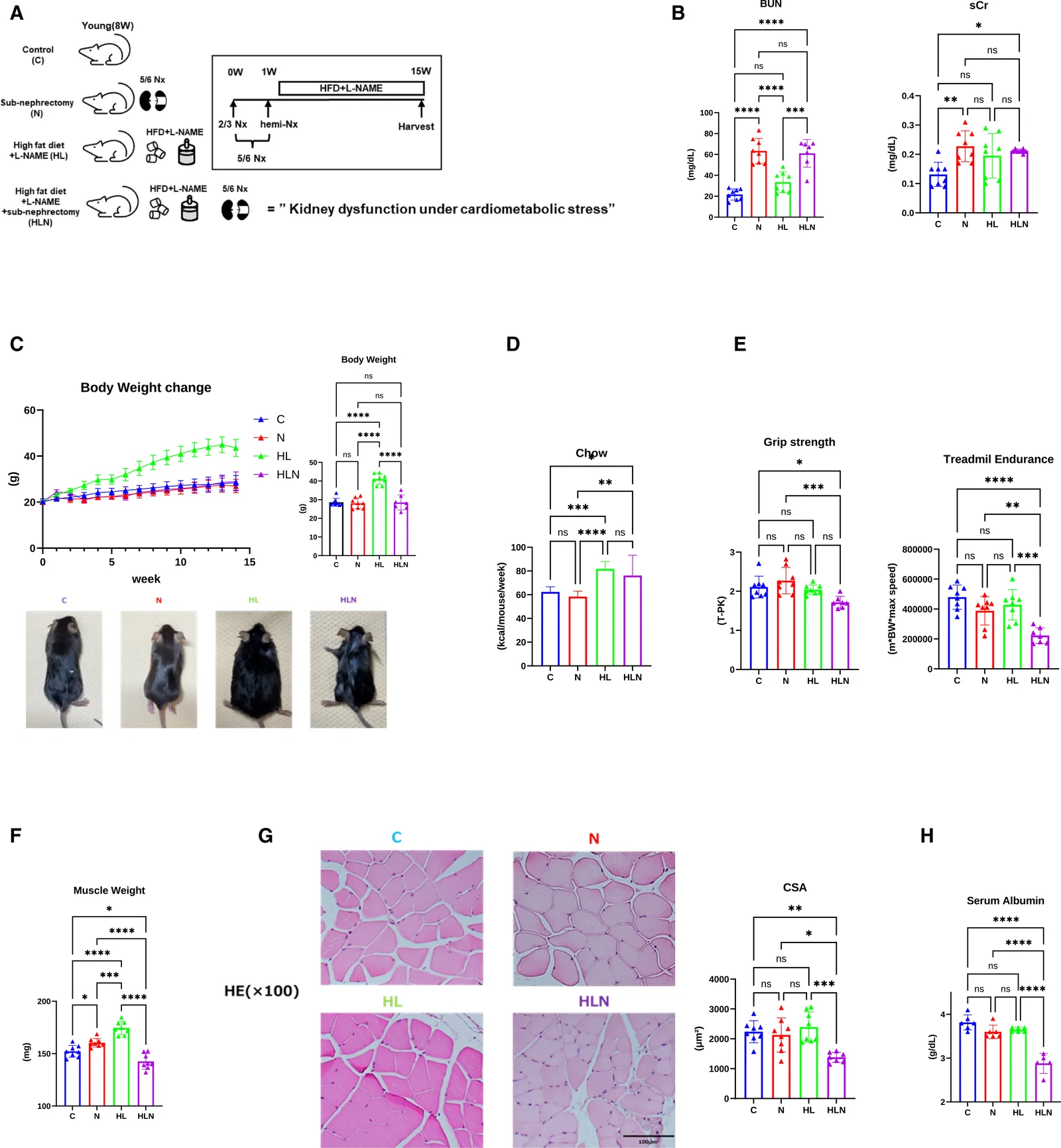
Kidney dysfunction under cardiometabolic stress is associated with calorie-intake-independent wasting and frailty-like muscle phenotypes (A) Experimental design and group allocation for control (C), nephrectomized (N), high-fat/L-NAME-treated (HL), and high-fat/L-NAME-treated nephrectomized (HLN) mice. (B) Blood urea nitrogen and serum creatinine levels. N and HLN mice exhibited impaired renal function (C, *n* = 8; N, *n* = 8; HL, *n* = 8; HLN, *n* = 7). (C) Body weight trajectory and body weight at sacrifice. HL mice showed marked weight gain, whereas HLN mice failed to gain weight during the observation period and showed reduced body weight at sacrifice (C, *n* = 8; N, *n* = 8; HL, *n* = 8; HLN, *n* = 7). (D) Caloric intake. Caloric intake was comparable between HL and HLN mice (C, *n* = 8; N, *n* = 8; HL, *n* = 8; HLN, *n* = 7). (E) Grip strength and treadmill endurance. HLN mice exhibited impaired muscle strength and exercise capacity (C, *n* = 8; N, *n* = 8; HL, *n* = 8; HLN, *n* = 7). (F) Gastrocnemius muscle weight. HLN mice showed reduced skeletal muscle mass (C, *n* = 8; N, *n* = 8; HL, *n* = 8; HLN, *n* = 7). (G) Representative histological images of skeletal muscle and quantification of myofiber cross-sectional area. HLN mice exhibited reduced myofiber size. Scale bars, 100 μm. (C, *n* = 8; N, *n* = 8; HL, *n* = 8; HLN, *n* = 7). (H) Serum albumin levels. HLN mice exhibited lower serum albumin levels. (C, *n* = 8; N, *n* = 8; HL, *n* = 8; HLN, *n* = 7). Data are reported as mean ± SD. Exact *n* values for each experimental group are indicated in the corresponding images or figure legend. Statistical analyses were performed using one-way ANOVA followed by Tukey’s multiple-comparison test. Significance was defined as ∗*p* < 0.05, ∗∗*p* < 0.01, ∗∗∗*p* < 0.001, and ∗∗∗∗*p* < 0.0001.

HL mice showed marked weight gain during the observation period, whereas HLN mice failed to gain weight and exhibited lower body weight at sacrifice despite caloric intake comparable to that of HL mice (Figures 1C and 1D). Thus, nephron loss in the setting of HFD + L-NAME exposure was associated with calorie-intake-independent wasting. Consistent with this phenotype, HLN mice exhibited reduced grip strength, impaired treadmill endurance, decreased gastrocnemius muscle weight, reduced myofiber cross-sectional area, and lower serum albumin levels (Figures 1E–1H).

We next examined whether this phenotype could be explained by differences in cardiovascular stress or systemic inflammation. Consistent with previous reports,^39^ HFD + L-NAME increased blood pressure and induced HFpEF-like cardiac features, including preserved systolic function and increased E/e’. However, these cardiovascular features were observed in both HL and HLN mice and were not further exacerbated by nephron loss (Figures S1A–S1C). Because systemic inflammation may also contribute to CKD-associated wasting, we measured circulating inflammatory cytokines. Serum IL-6 levels did not differ significantly among groups, whereas serum TNFα levels were increased in nephrectomized mice but did not uniquely distinguish HLN mice from N mice (Figures S1D and S1E). Thus, the selective development of frailty-like muscle phenotypes in HLN mice was not readily explained by HLN-specific worsening of cardiovascular stress or systemic cytokine elevation.

Despite comparable exposure to high-fat feeding, HL and HLN mice showed divergent tissue responses. Serum total cholesterol levels were increased in both HL and HLN mice, whereas overt hepatic steatosis was observed in HL mice but not in HLN mice (Figures S1F and S1G). These findings indicate that HLN mice were exposed to high-fat feeding, as reflected by increased serum total cholesterol levels, but did not develop the obesity-prone and steatotic phenotype observed in HL mice. Instead, superimposed nephron loss was associated with a shift toward impaired weight gain, skeletal muscle wasting, and hypoalbuminemia under cardiometabolic stress.

To further define the contribution of each component of the HLN model, we compared nephrectomy alone (N), nephrectomy plus L-NAME (LN), nephrectomy plus high-fat diet (HN), and nephrectomy plus high-fat diet and L-NAME (HLN) mice (Figure S2A). Renal dysfunction was comparable among these nephrectomized groups (Figures S2B and S2C). Although HN mice showed marked weight gain under high-fat feeding, HLN mice exhibited blunted weight gain despite comparable caloric intake (Figure S2D). Frailty-like muscle phenotypes, including reduced grip strength, impaired treadmill endurance, lower gastrocnemius muscle weight, smaller myofiber cross-sectional area, and lower serum albumin levels, were evident only in HLN mice (Figures S2E–S2I). Thus, the full HLN phenotype was observed when nephron loss was combined with both high-fat feeding and L-NAME treatment. Together, these findings show that HLN mice develop calorie-intake-independent wasting and frailty-like muscle phenotypes under cardiometabolic stress.

### Superimposed nephron loss attenuates cardiometabolic stress-induced glucose intolerance and hyperinsulinemia

Because HLN mice failed to gain body weight and developed skeletal muscle wasting despite preserved caloric intake, we next asked whether this phenotype was associated with altered whole-body glucose handling. As cardiometabolic stress is commonly associated with glucose intolerance and compensatory hyperinsulinemia, we first performed intraperitoneal glucose tolerance tests (IPGTTs). Nephrectomized mice showed a modest increase in blood glucose excursion relative to control mice, whereas HL mice exhibited a more pronounced increase. In contrast, blood glucose excursion in HLN mice was attenuated relative to HL mice, and the AUC did not differ significantly from that of control mice (Figures 2A and 2B).

**Figure 2:**
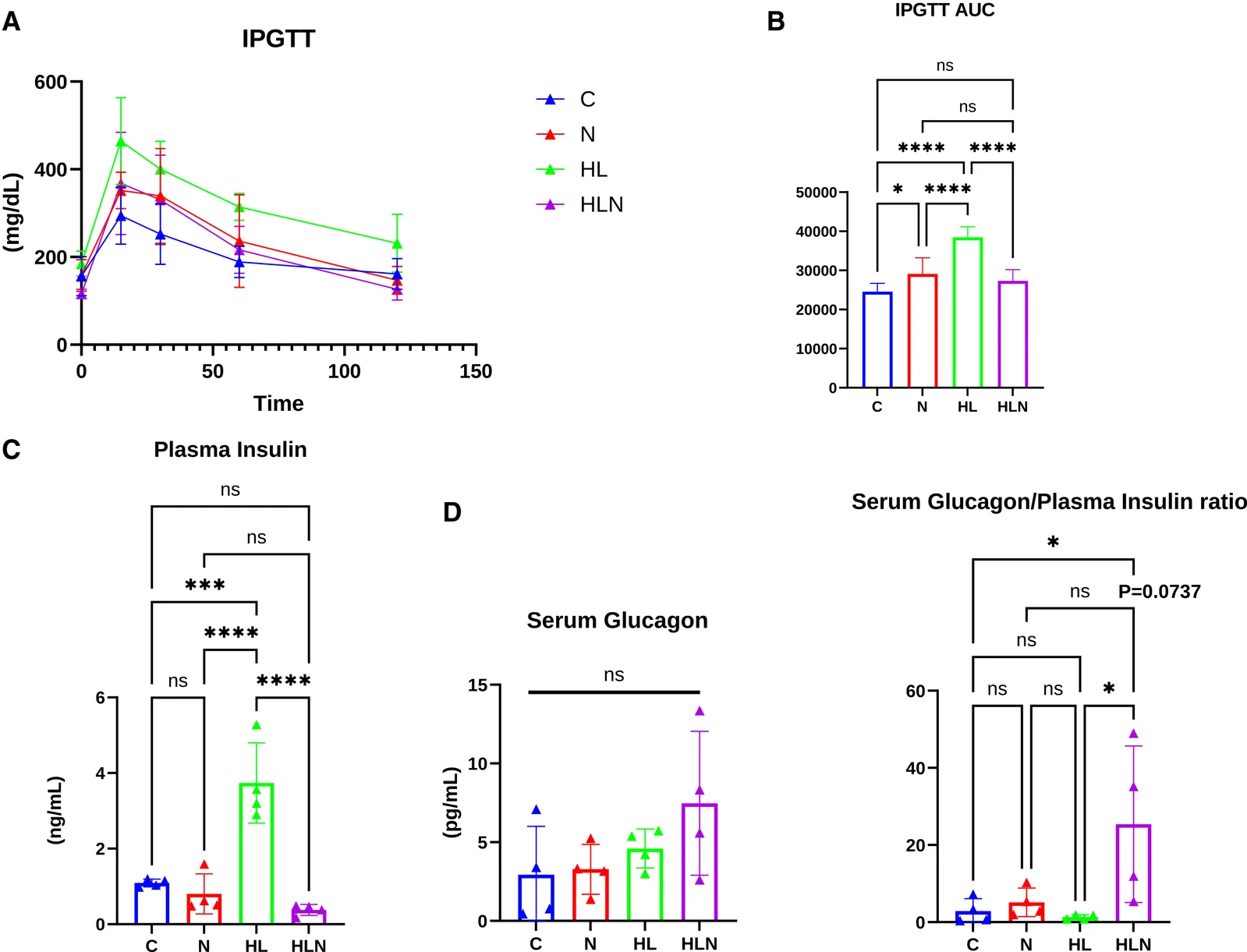
Superimposed nephron loss attenuates cardiometabolic stress-induced glucose intolerance and fasting hyperinsulinemia (A) Blood glucose levels during the intraperitoneal glucose tolerance test (IPGTT). HL mice showed impaired glucose tolerance, whereas blood glucose excursion in HLN mice was attenuated relative to HL mice. (C, *n* = 8; N, *n* = 8; HL, *n* = 8; HLN, *n* = 6). (B) Quantification of the area under the curve (AUC) for the IPGTT shown in (A). The AUC in HLN mice did not differ significantly from that in control mice. (C, *n* = 8; N, *n* = 8; HL, *n* = 8; HLN, *n* = 6). (C) Fasting plasma insulin levels. Fasting insulin levels were markedly elevated in HL mice but remained comparable to control levels in HLN mice. (C, *n* = 4; N, *n* = 4; HL, *n* = 4; HLN, *n* = 4). (D) Fasting serum glucagon levels and glucagon-to-insulin ratio. Although fasting serum glucagon levels did not differ significantly among groups, the glucagon-to-insulin ratio was increased in HLN mice. (C, *n* = 4; N, *n* = 4; HL, *n* = 4; HLN, *n* = 4). Data are reported as the mean ± SD. Exact *n* values for each experimental group are indicated in the corresponding images or figure legend. Statistical analyses were performed using one-way ANOVA followed by Tukey’s multiple-comparison test. Significance was defined as ∗*p* < 0.05, ∗∗*p* < 0.01, ∗∗∗*p* < 0.001, and ∗∗∗∗*p* < 0.0001.

Consistent with this pattern, fasting insulin levels were markedly elevated only in HL mice, whereas insulin levels in HLN mice remained comparable to those in control mice (Figure 2C). Fasting glucagon levels did not differ significantly among the groups, although several HLN mice showed higher glucagon levels. Consequently, the glucagon-to-insulin ratio was increased in HLN mice (Figure 2D), indicating a hormonal profile distinct from the fasting hyperinsulinemia observed in HL mice. Thus, superimposed nephron loss attenuated the glucose-intolerant and fasting hyperinsulinemic phenotype induced by HFD + L-NAME treatment. These findings indicate that the wasting phenotype in HLN mice was not accompanied by the overt glucose intolerance or fasting hyperinsulinemia observed in HL mice.

### HLN mice exhibit skeletal muscle metabolic remodeling without sustained induction of canonical atrophy genes

Although superimposed nephron loss attenuated the glucose-intolerant phenotype induced by HFD + L-NAME treatment, HLN mice developed marked skeletal muscle wasting. We first examined skeletal muscle carbohydrate storage in the HLN model. After 12 h fasting, skeletal muscle glycogen content was reduced in N and HLN mice compared with C and HL mice (Figure 3A). This reduction prompted us to examine whether glucose-related metabolic programs were altered in skeletal muscle. We then performed integrated transcriptomic and metabolomic analyses to define the metabolic programs associated with the HLN skeletal muscle phenotype.

**Figure 3:**
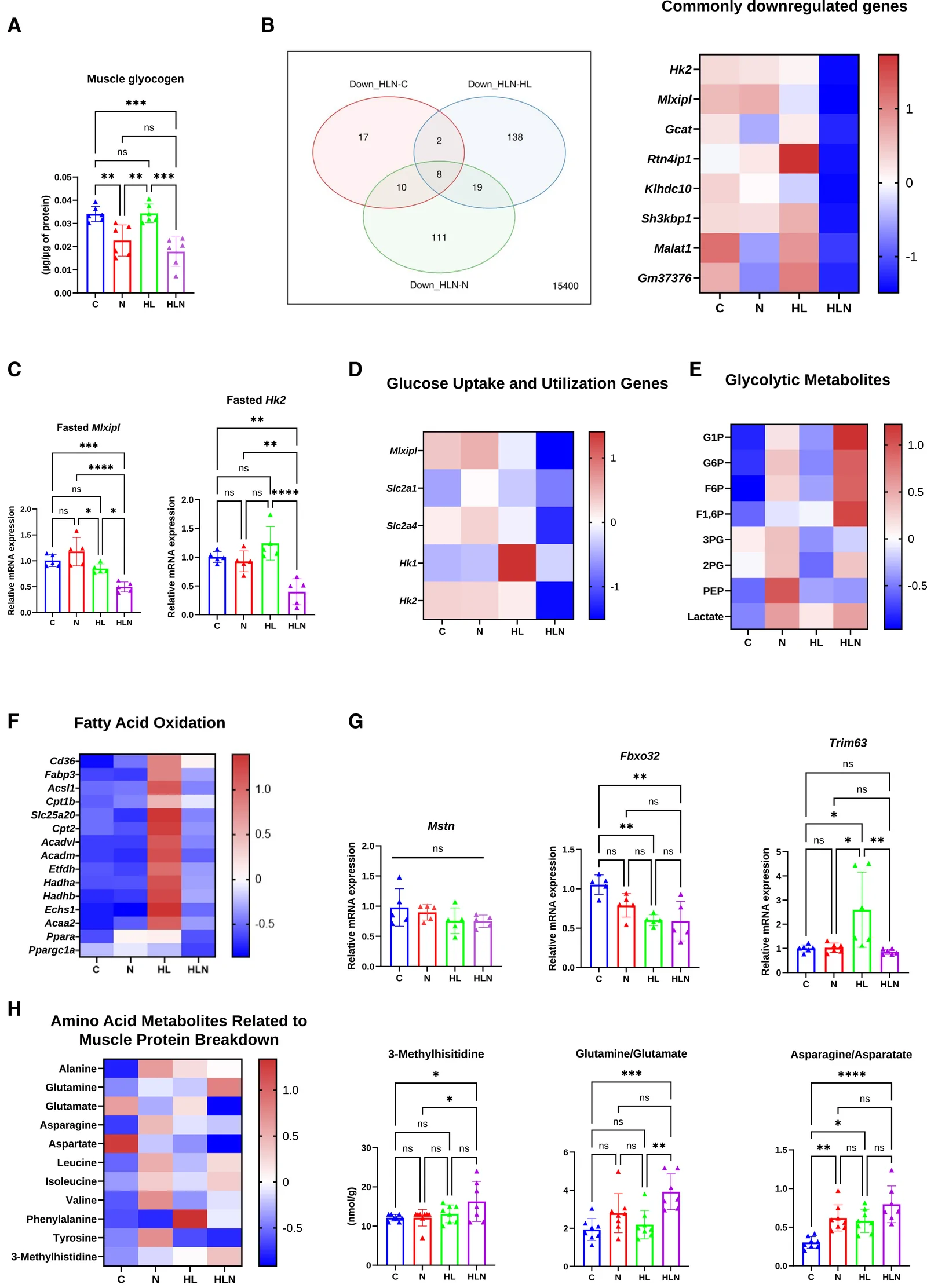
HLN mice exhibit skeletal muscle metabolic remodeling without sustained induction of canonical atrophy genes (A) Skeletal muscle glycogen content after 12 h fasting. Skeletal muscle glycogen content was reduced in N and HLN mice. (C, *n* = 6; N, *n* = 6; HL, *n* = 6; HLN, *n* = 6). (B) Venn diagram of commonly downregulated genes in HLN skeletal muscle across comparisons with C, N, and HL mice. Eight genes were commonly downregulated in HLN mice, including *Hk2* and *Mlxipl*. (C, *n* = 3; N, *n* = 3; HL, *n* = 3; HLN, *n* = 3). (C) Expression of *Hk2* and *Mlxipl* under fasted conditions. (C, *n* = 5; N, *n* = 5; HL, *n* = 5; HLN, *n* = 5). (D) Heatmap of selected glucose-related genes in skeletal muscle. HLN mice showed reduced expression of genes related to glucose sensing, glucose transport, and glycolysis. (C, *n* = 3; N, *n* = 3; HL, *n* = 3; HLN, *n* = 3). (E) Heatmap of glycolytic metabolites in skeletal muscle. HLN mice showed accumulation of glycolytic metabolites. (C, *n* = 3; N, *n* = 3; HL, *n* = 3; HLN, *n* = 3). (F) Heatmap of selected fatty acid oxidation-related genes in skeletal muscle. HLN mice showed reduced expression compared to HL. (C, *n* = 3; N, *n* = 3; HL, *n* = 3; HLN, *n* = 3). (G) Expression of classical atrophy-related genes, including *Mstn*, *Fbxo32*, and *Trim63*. These genes were not increased in HLN skeletal muscle. (C, *n* = 5; N, *n* = 5; HL, *n* = 5; HLN, *n* = 5). (H) Metabolites and metabolite ratios related to amino acid catabolism and nitrogen metabolic remodeling. HLN mice showed amino acid catabolic signatures, including increased 3-methylhistidine and altered amino acid-related metabolic ratios. (C, *n* = 8; N, *n* = 8; HL, *n* = 8; HLN, *n* = 7). Data are reported as the mean ± SD. Exact *n* values for each experimental group are indicated in the corresponding images or figure legend. Statistical analyses were performed using one-way ANOVA followed by Tukey’s multiple-comparison test. Significance was defined as ∗*p* < 0.05, ∗∗*p* < 0.01, ∗∗∗*p* < 0.001, and ∗∗∗∗*p* < 0.0001.

Venn diagram analysis of differentially expressed genes identified eight genes that were commonly downregulated in HLN skeletal muscle across comparisons with C, N, and HL mice: *Hk2, Mlxipl, Gcat, Rtn4ip1, Klhdc10, Sh3kbp1, Malat1,* and *Gm37376*—whereas no commonly upregulated genes were detected under the same criteria (Figures 3B and S3A–S3C). This common downregulated gene set included *Hk2*, a key glycolytic enzyme, and *Mlxipl,* encoding the glucose-responsive transcription factor *ChREBP*, together with genes linked to amino acid metabolism, mitochondrial or stress-related pathways, muscle development, and non-coding RNA regulation. GO enrichment analysis of genes altered in HLN skeletal muscle relative to C mice identified glucose- and carbohydrate-related molecular functions among the enriched terms (Figure S3D). Based on these transcriptomic findings, we focused on *Hk2* and *Mlxipl* as glucose-related genes emerging from the HLN skeletal muscle signature and examined their expression under fed and fasted conditions. *Hk2* and *Mlxipl* were reduced in HLN mice under fasting conditions, whereas these differences were less evident under fed conditions (Figures 3C and S3E). These findings indicate that fasting selectively reveals suppression of glucose-related transcriptional programs in HLN skeletal muscle.

Consistent with these findings, a heatmap of selected genes involved in glucose sensing, glucose transport, and glycolysis showed reduced expression of key glucose-utilization genes in HLN skeletal muscle, including *Mlxipl, Slc2a4,* and *Hk2*(Figures 3D and S3F). Metabolite profiling further showed accumulation of glycolytic intermediates in HLN skeletal muscle (Figure 3E). Together, the lower-expression pattern of glucose-related transcriptional programs and accumulation of glycolytic metabolites were consistent with disrupted handling of glucose-derived substrates in HLN skeletal muscle, despite the absence of the glucose-intolerant phenotype observed in HL mice.

Given that fatty acids are another major energy substrate for skeletal muscle, we next examined fatty acid metabolism-related genes. HL mice showed upregulation of fatty acid oxidation- and lipid metabolism-related genes, whereas this HL-associated induction was not observed in HLN mice despite high-fat feeding (Figure 3F). KEGG pathway enrichment analysis of genes downregulated in HLN relative to HL further identified fatty acid degradation and fatty acid metabolism among the top enriched pathways (Figure S3G). These findings suggest that HLN skeletal muscle fails to mount the lipid-utilization gene program observed in HL mice under high-fat feeding.

To determine whether these metabolic alterations were accompanied by sustained activation of canonical muscle atrophy programs, we examined the expression of classical atrophy-related genes, including *Mstn*, *Fbxo32*, and *Trim63*. These genes were not increased in HLN mice, indicating that sustained activation of canonical transcriptional atrophy programs was not evident (Figure 3G). Nevertheless, metabolomic signatures, including increased *3-methylhistidine* and altered amino acid ratios, were consistent with amino acid catabolism and nitrogen metabolic remodeling (Figure 3H). Together, these findings suggest that HLN-associated muscle wasting is less likely to reflect sustained activation of canonical transcriptional atrophy programs, and instead reflects a metabolically maladaptive state characterized by disrupted glucose-related programs and failure to induce fatty acid utilization-related programs under high-fat feeding. The accompanying increase in *3-methylhistidine* and altered amino acid ratios further suggests increased amino acid mobilization and nitrogen metabolic remodeling.

### HLN mice exhibit impaired glycemic response to gluconeogenic substrates and hepatic metabolic remodeling

The skeletal muscle findings suggested altered carbohydrate storage and glucose-related metabolic adaptation in HLN mice. We next assessed the systemic glycemic response to gluconeogenic substrates using the lactate-pyruvate tolerance test (LPTT). Because fasting glucose levels at 0 min did not differ significantly among groups (Figure S4A; absolute glucose concentrations), we analyzed change-from-baseline glucose excursions to evaluate the response to lactate/pyruvate loading (Figure 4A; Δglucose). Whereas N and HL mice showed greater glucose excursions after lactate-pyruvate administration than control mice, HLN mice exhibited a blunted glycemic response, with significantly lower Δglucose AUC (Figures 4A and 4B).

**Figure 4:**
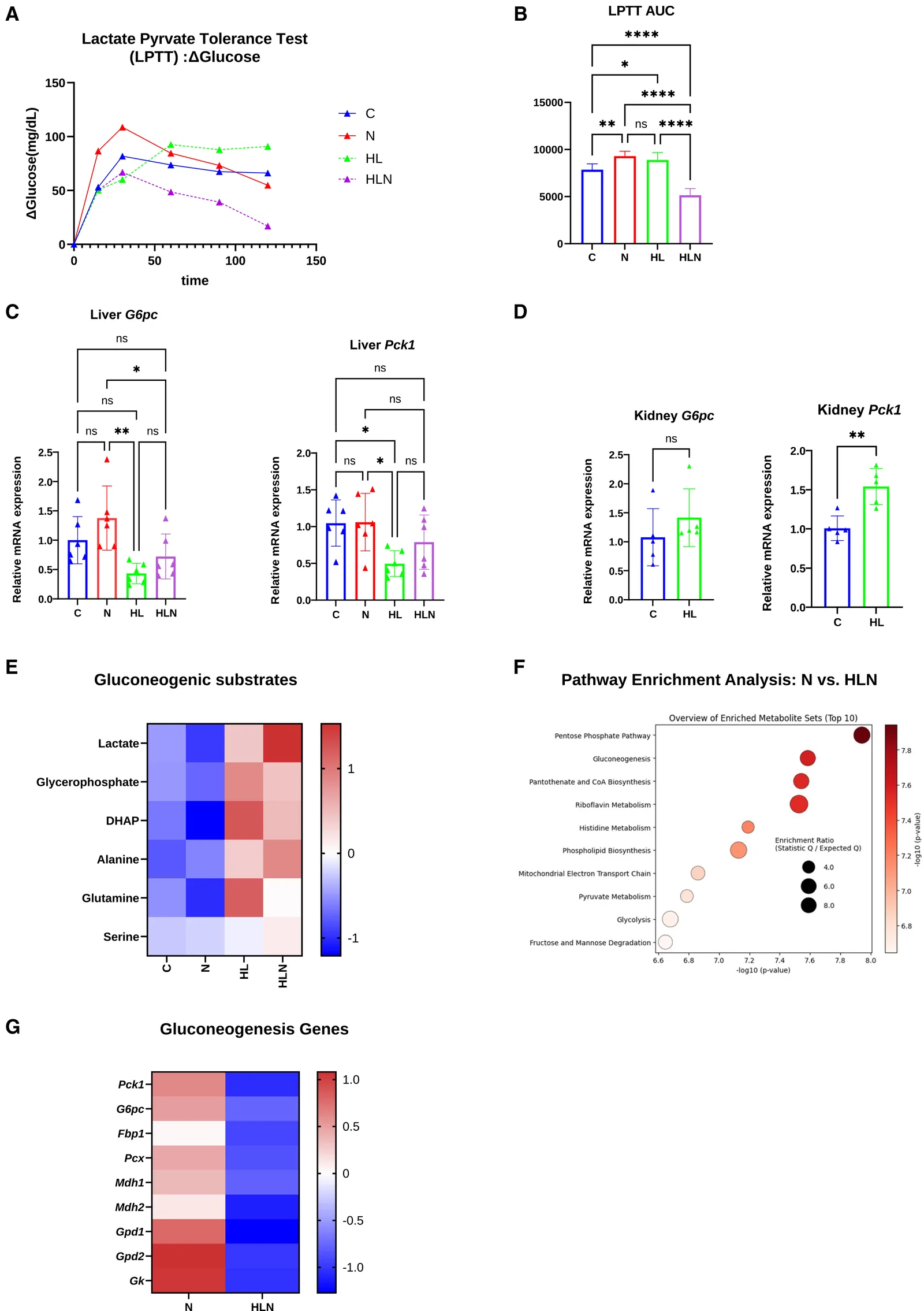
HLN mice exhibit impaired glycemic response to gluconeogenic substrates and hepatic metabolic remodeling (A) ΔBlood glucose levels during the lactate-pyruvate tolerance test (LPTT). HLN mice exhibited a blunted glycemic response after lactate-pyruvate administration. (C, *n* = 8; N, *n* = 8; HL, *n* = 7; HLN, *n* = 8). (B) Quantification of the area under the curve (AUC) for the LPTT shown in (A). (C, *n* = 8; N, *n* = 8; HL, *n* = 7; HLN, *n* = 8). (C) Hepatic expression of *Pck1* and *G6pc*. Hepatic *Pck1* and *G6pc* expression was markedly reduced in HL mice and partially restored in HLN mice, although not to control levels. (C, *n* = 5; N, *n* = 5; HL, *n* = 5; HLN, *n* = 5). (D) Renal expression of *Pck1* and *G6pc*. Renal *Pck1* expression tended to increase under HL conditions. (C, *n* = 5; HL, *n* = 5). (E) Heatmap or relative abundance of selected hepatic metabolites related to gluconeogenesis and glucose-related metabolic remodeling. Gluconeogenesis-related substrates and intermediates accumulated in HL and HLN mice relative to C and N mice. (C, *n* = 8; N, *n* = 8; HL, *n* = 8; HLN, *n* = 7). (F) Metabolite enrichment analysis of HLN livers relative to N mice. The pentose phosphate pathway was the most significantly enriched pathway, followed by gluconeogenesis-related pathways. (N, *n* = 8; HLN, *n* = 7). (G) Heatmap of selected hepatic metabolic genes, including gluconeogenesis-related genes. HLN livers showed downregulation of selected hepatic metabolic genes relative to N mice within the two-group comparison. (N, *n* = 3; HLN, *n* = 3). Data are reported as mean ± SD. Exact *n* values for each experimental group are indicated in the corresponding images or figure legend. Statistical analyses were performed using one-way ANOVA followed by Tukey’s multiple-comparison test. Significance was defined as ∗*p* < 0.05, ∗∗*p* < 0.01, ∗∗∗*p* < 0.001, and ∗∗∗∗*p* < 0.0001.

To further characterize gluconeogenic pathways in relation to the impaired glycemic response observed in the LPTT, we examined the expression of key gluconeogenic enzymes in the liver and kidney. In the liver, *Pck1* and *G6pc* expression was markedly reduced in HL mice, whereas both genes showed partial recovery in HLN mice relative to HL mice, although not to control levels (Figure 4C). In the kidney, *Pck1* expression was increased under HL conditions (Figure 4D). Although these data do not establish organ-specific glucose output, they indicate altered regulation of hepatic and renal gluconeogenic pathways under cardiometabolic stress.

We then examined broader hepatic metabolic remodeling using metabolomic and transcriptomic analyses. Principal component analysis showed that hepatic metabolomic profiles of HL and HLN mice were largely overlapping and clearly separated from those of C and N mice, suggesting that a major component of hepatic metabolic remodeling was associated with HFD + L-NAME-induced cardiometabolic stress rather than nephron loss alone (Figure S4B). Consistent with this pattern, hepatic metabolite profiling showed accumulation of gluconeogenesis-related substrates and intermediates in both HL and HLN mice relative to C and N mice (Figures 4E and S4C). Metabolite enrichment analysis of HLN livers relative to N mice identified the pentose phosphate pathway as the most significantly enriched pathway, followed by gluconeogenesis-related pathways (Figure 4F). RNA sequencing further showed coordinated downregulation of a selected gluconeogenesis-related gene program, including *Pck1*, *G6pc*, *Fbp1*, and *Pcx*, in HLN livers relative to N mice and across the two-group heatmap (Figure 4G).

Together, these analyses indicate that HFD + L-NAME-induced cardiometabolic stress was associated with hepatic glucose-related metabolic remodeling in both HL and HLN mice, including altered gluconeogenesis-related metabolites and genes, despite the absence of overt hepatic steatosis in HLN mice. In contrast, only HLN mice exhibited a blunted glycemic response to lactate-pyruvate challenge, suggesting that superimposed nephron loss compromises adaptive glucose homeostasis under cardiometabolic stress.

### Aging and nephron loss are associated with frailty-like muscle phenotypes and impaired glycemic response

Having defined a wasting phenotype associated with skeletal muscle metabolic remodeling and impaired glycemic response in HLN mice, we next asked whether similar frailty-related and glycemic alterations were observed in an aging-associated context. We analyzed young and aged mice with or without subnephrectomy, generating four groups: young control (YC), young nephrectomized (YN), aged control (AC), and aged nephrectomized (AN) mice (Figure 5A). Blood urea nitrogen and serum creatinine levels were increased in aged nephrectomized mice, consistent with impaired renal function after subnephrectomy (Figures S5A and S5B). Caloric intake was not significantly reduced by nephron loss in aged mice (Figure S5C).

**Figure 5:**
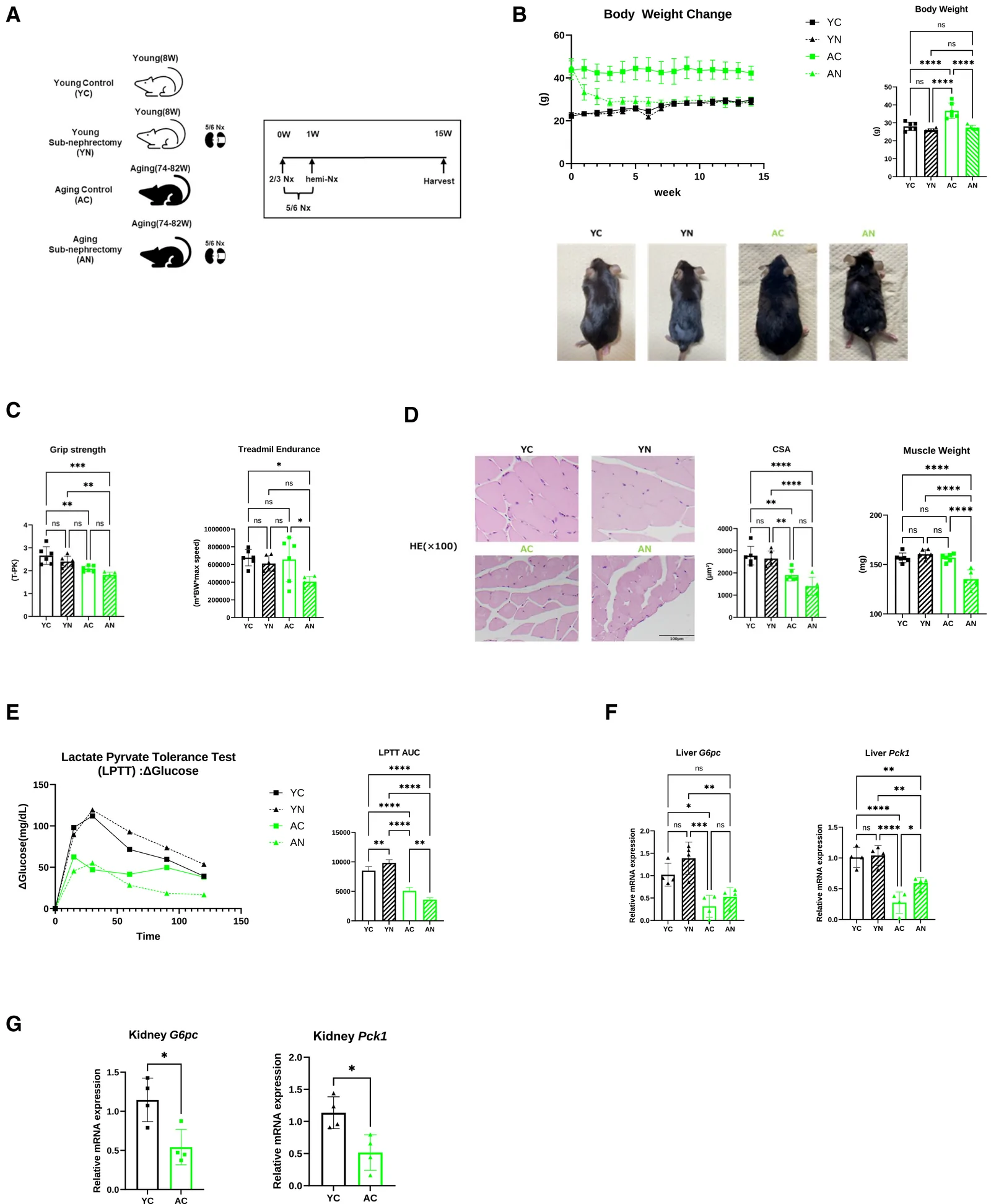
Aging and nephron loss are associated with frailty-like muscle phenotypes and impaired glycemic response (A) Experimental design and group allocation for young control (YC), young nephrectomized (YN), aged control (AC), and aged nephrectomized (AN) mice. (B) Body weight trajectory and body weight at sacrifice. AN mice exhibited sustained body weight loss after nephron loss and failed to regain weight during the observation period. (YC, *n* = 6; YN, *n* = 6; AC, *n* = 6; AN, *n* = 5). (C) Grip strength and treadmill endurance. Grip strength was reduced with aging, whereas treadmill endurance was markedly impaired when nephron loss was superimposed on aging. (YC, *n* = 6; YN, *n* = 6; AC, *n* = 6; AN, *n* = 5). (D) Gastrocnemius muscle weight and myofiber cross-sectional area. Gastrocnemius muscle weight was low only in aged nephrectomized mice, whereas myofiber size was reduced by aging and further decreased by nephrectomy. Scale bars, 100 μm. (YC, *n* = 6; YN, *n* = 6; AC, *n* = 6; AN, *n* = 5). (E) ΔBlood glucose levels during the lactate-pyruvate tolerance test (LPTT) and corresponding AUC. Aging attenuated the glycemic response, and nephron loss further exacerbated this impairment in aged mice. (YC, *n* = 5; YN, *n* = 5; AC, *n* = 5; AN, *n* = 5). (F) Hepatic expression of *Pck1* and *G6pc*. Aging reduced the expression of key hepatic gluconeogenic enzymes. (YC, *n* = 4; YN, *n* = 4; AC, *n* = 4; AN, *n* = 4). (G) Renal expression of *Pck1* and *G6pc*. Aging reduced the expression of key renal gluconeogenic enzymes. (YC, *n* = 4; AC, *n* = 4). Data are reported as mean ± SD. For (B)–(D), YC, YN, and AC groups included six mice, and the AN group included five mice. For the LPTT in E, five mice were analyzed per group. Exact *n* values for gene-expression analyses are indicated for the corresponding images. Statistical analyses were performed using one-way ANOVA followed by Tukey’s multiple-comparison test. Significance was defined as ∗*p* < 0.05, ∗∗*p* < 0.01, ∗∗∗*p* < 0.001, and ∗∗∗∗*p* < 0.0001.

Young nephrectomized mice did not exhibit overt frailty-like muscle phenotypes during the observation period. In contrast, aged nephrectomized mice showed sustained body weight loss after nephron loss and failed to regain weight thereafter (Figure 5B). In parallel, grip strength was reduced with aging, whereas treadmill endurance was markedly impaired when nephron loss was superimposed on aging (Figure 5C). Gastrocnemius muscle weight was low only in aged nephrectomized mice, and myofiber cross-sectional area was reduced by aging (Figure 5D). These findings indicate that, similar to the HLN model under cardiometabolic stress, nephron loss superimposed on aging is associated with calorie-intake-independent wasting and frailty-like muscle phenotypes.

We next assessed systemic glycemic responses to lactate/pyruvate loading using the LPTT after a 16-h fast. Absolute glucose concentrations are shown in Figure S5D, whereas change-from-baseline glucose excursions and AUC are shown in Figure 5E. Aged control mice exhibited blunted glucose excursions compared with young control mice. Nephron loss further attenuated this response in aged mice, whereas its effect was limited in young mice (Figure 5E). In parallel, hepatic and renal expression of *Pck1* and *G6pc* was reduced in aged mice relative to young controls (Figures 5F and 5G). In aged nephrectomized mice, hepatic *Pck1* expression was increased compared with aged controls but remained lower than in young mice, consistent with an adaptive increase in hepatic *Pck1* expression in the setting of reduced renal mass. Serum insulin and glucagon levels were not significantly altered by aging or nephrectomy (Figures S5E–S5G), indicating that these changes were not accompanied by overt differences in fasting endocrine profiles.

Together, these findings show that nephron loss superimposed on aging recapitulates key features observed in HLN mice, including a more pronounced frailty-like phenotype and impaired glycemic response to lactate-pyruvate challenge, accompanied by reduced hepatic and renal gluconeogenic gene expression.

### Renal PCK1 expression is reduced in older patients with CKD

We further examined whether age-associated changes in renal glucose-related metabolic machinery were evident in human CKD tissue. We analyzed renal phosphoenolpyruvate carboxykinase 1 (*PCK1*) expression in kidney biopsy specimens from patients with CKD. Among 210 patients diagnosed with nephrosclerosis between 2018 and 2025, individuals with relatively preserved renal function, defined as serum creatinine ≤ 2.0 mg/dL, were selected to reduce potential confounding by advanced renal impairment (Figure 6A). After excluding patients with diabetes, defined as HbA1c ≥ 7.0%, and samples with insufficient cortical tissue, 30 individuals remained for analysis, including 14 younger patients aged ≤50 years and 16 older patients aged ≥75 years. Clinical characteristics are summarized in Table 1. As expected, age differed significantly between the groups, whereas estimated glomerular filtration rate, body mass index, proteinuria, serum albumin, and glycemic indices were comparable.

**Figure 6:**
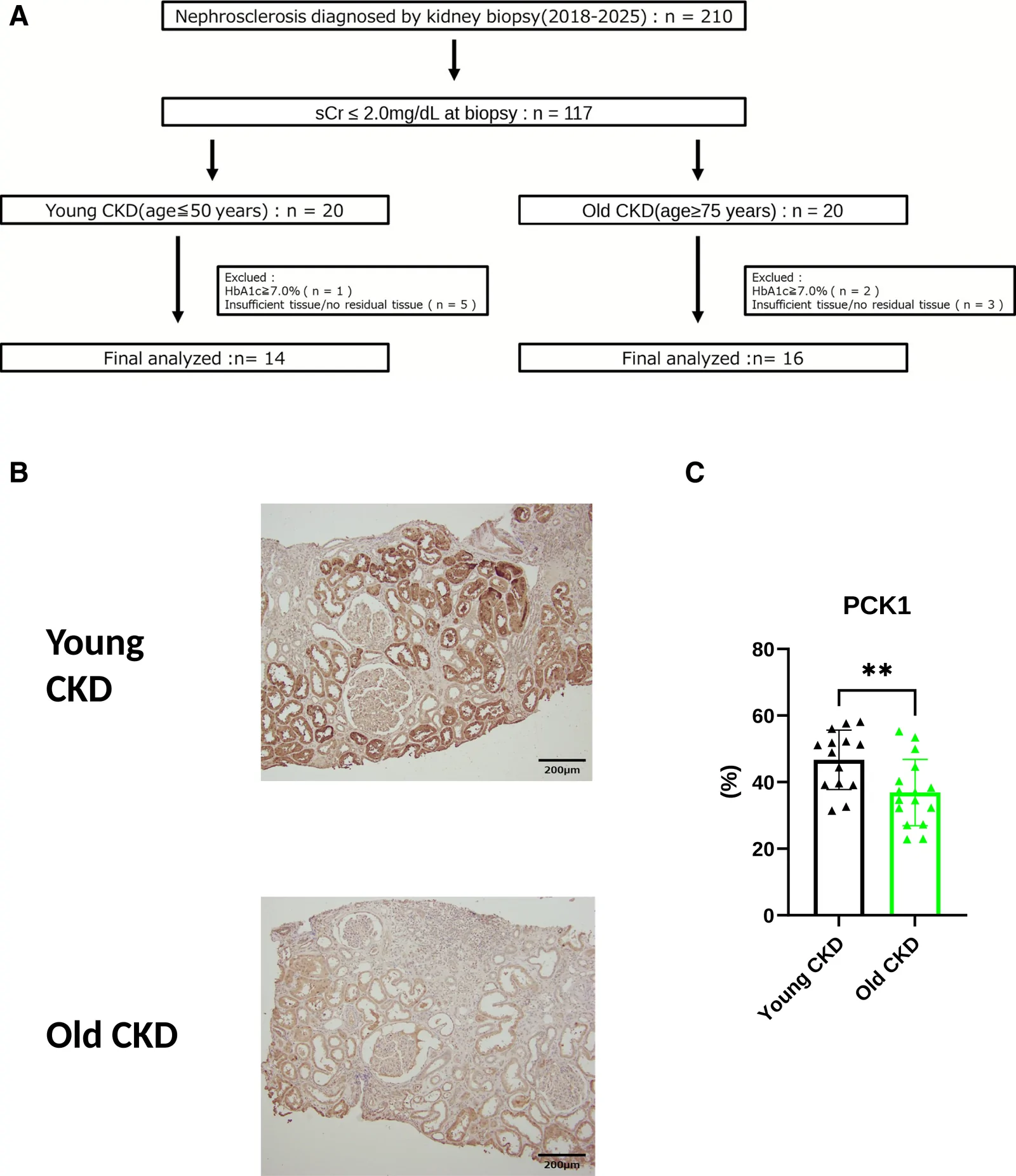
Renal PCK1 expression is reduced in older patients with CKD (A) Flowchart of patient selection for analysis of renal *PCK1* expression in kidney biopsy specimens from patients with CKD. (B) Representative images of *PCK1* immunostaining in kidney biopsy specimens from younger and older patients with CKD. *PCK1* expression was detected predominantly in renal tubular cells and was reduced in older patients. Scale bars, 200 μm. (C) Quantification of *PCK1*-positive tubular area in kidney biopsy specimens from younger and older patients with CKD. Older patients exhibited a lower *PCK1*-positive tubular area than younger patients (young CKD, *n* = 14; old CKD, *n* = 16). Clinical characteristics of the study cohort are summarized in Table 1. Data are reported as the mean ± SD. The younger and older groups included 14 and 16 participants, respectively. Statistical analysis was performed using the Mann-Whitney *U* test. Significance was defined as ∗∗*p* < 0.01.

**Table 1:** Baseline characteristics of the young and old groups

	Young CKD (*n* = 14)	Old CKD (*n* = 16)	*p* value
Age, years	40.00 ± 8.41	78.14 ± 2.45	<0.0001
Sex, male (%)	71.43	81.25	0.6746
BMI, kg/m^2^	25.90 ± 4.82	23.00 ± 2.90	0.0633
eGFR, mL/min/1.73m^2^	48.61 ± 13.87	40.76 ± 12.34	0.7068
Creatinine, mg/dL	1.31 ± 0.26	1.37 ± 0.33	0.5704
Up/Ucr, g/g・Cr	2.27 ± 3.81	2.10 ± 1.97	0.883
Na-Cl, mEq/L	36.51 ± 2.40	34.44 ± 3.18	0.052
Albumin, g/dL	4.05 ± 0.74	3.78 ± 0.44	0.2629
HbA1c (%)	5.66 ± 0.53	5.89 ± 0.62	0.2947
Glu, mg/dL	98.33 ± 12.75	118.7 ± 41.09	0.0867

Data are reported as the mean ± standard deviation (SD), and *p* values were calculated using unpaired two-tailed Student’s *t* tests or chi-square tests for categorical variables. BMI, body mass index; CKD, chronic kidney disease; eGFR, estimated glomerular filtration rate; Up/UCr, urinary protein-to-creatinine ratio.

In kidney biopsy specimens from older patients, many renal tubules showed weak or undetectable *PCK1* staining (Figure 6B). Quantitative analysis demonstrated that the *PCK1*-positive tubular area was significantly reduced in older patients compared with younger patients (Figure 6C). These findings are consistent with an age-associated reduction in renal tubular *PCK1* expression in human CKD.

### Nutritional modulation with cornstarch ameliorates frailty-like muscle phenotypes in HLN mice

The physiological and omics findings suggested that HLN mice exhibit fasting-associated suppression of skeletal muscle glucose-related metabolic programs together with a blunted glycemic response to gluconeogenic substrates. Because slowly digestible cornstarch is used to support glycemic maintenance during fasting intervals in disorders characterized by impaired fasting glucose homeostasis, we examined whether cornstarch-based nutritional modulation could buffer fasting-associated glycemic vulnerability and mitigate sarcopenic and physical frailty features in HLN mice (Figure 7A). Cornstarch feeding increased blood glucose levels after 8 h fasting, whereas this difference was no longer evident after 12 h fasting, and renal function was not significantly altered (Figures 7B, S6A and S6B). These findings suggest that cornstarch partially prolonged glycemic maintenance during fasting intervals without reversing kidney dysfunction.

**Figure 7:**
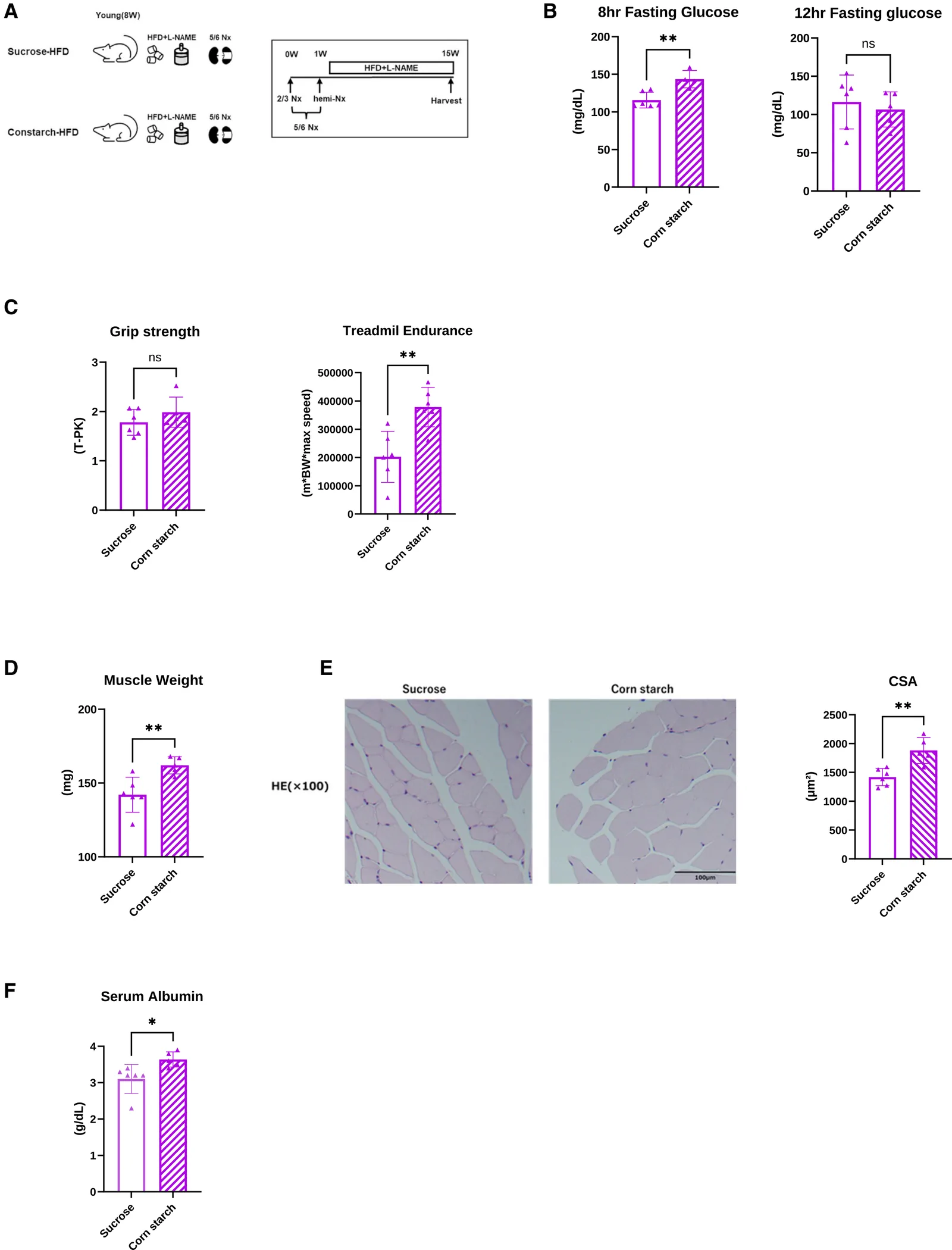
Nutritional modulation with cornstarch ameliorates frailty-like muscle phenotypes in HLN mice (A) Experimental design for nutritional modulation with sucrose-based or cornstarch-based diet in HLN mice. (B) Blood glucose levels after 8 h and 12 h fasting. Cornstarch feeding increased blood glucose levels after 8 h fasting, whereas this difference was no longer evident after 12 h fasting (sucrose, *n* = 6; corn starch, *n* = 6). (C) Grip strength and treadmill endurance. Cornstarch feeding improved treadmill endurance in HLN mice (sucrose, *n* = 6; corn starch, *n* = 6). (D) Gastrocnemius muscle weight. Cornstarch feeding increased gastrocnemius muscle mass in HLN mice (sucrose, *n* = 6; corn starch, *n* = 6). (E) Representative histological images of skeletal muscle and quantification of myofiber cross-sectional area. Cornstarch feeding increased myofiber size in HLN mice. Scale bars, 100 μm (sucrose, *n* = 6; corn starch, *n* = 6). (F) Serum albumin levels. Cornstarch feeding increased serum albumin levels in HLN mice (sucrose, *n* = 6; corn starch, *n* = 6). Data are reported as the mean ± SD. Six animals were analyzed per group. Statistical analyses were performed using unpaired two-tailed Student’s *t* tests. Significance was defined as ∗*p* < 0.05 and ∗∗*p* < 0.01.

Cornstarch-fed HLN mice showed lower body weight at sacrifice than sucrose-fed HLN mice (Figure S6C). However, this reduction in body weight was accompanied by increased gastrocnemius muscle mass and reduced epididymal and subcutaneous adipose tissue weights (Figures 7D and S6D), indicating a shift in body composition rather than generalized weight gain. In parallel, cornstarch-fed HLN mice exhibited improved treadmill endurance, increased myofiber cross-sectional area, and higher serum albumin levels (Figures 7C, 7E, and 7F). Cornstarch feeding also partially restored selected glucose-related genes under fasting conditions, including *Hk2*, whereas *Mlxipl* was not significantly increased (Figures S6E and S6F). These findings indicate that slowly digestible carbohydrate-based nutritional modulation ameliorates key frailty-like muscle phenotypes and hypoalbuminemia without reversing kidney dysfunction itself.

Because fatty acids are also major energy substrates for skeletal muscle, we additionally tested carnitine supplementation as an alternative metabolic intervention (Figure S7A). Although carnitine levels were reduced in CKD groups (Figure S7B), supplementation did not improve muscle function or mass and worsened glucose tolerance (Figures S7C–S7E). Together, these findings suggest that carbohydrate-based nutritional modulation may represent a useful strategy for mitigating frailty-like muscle phenotypes in this model.

## Discussion

In this study, we demonstrate in mouse models that superimposed kidney dysfunction redirects cardiometabolic stress from weight gain and glucose intolerance toward calorie-intake-independent wasting with sarcopenic and physical frailty features. This phenotypic switch was accompanied by metabolic remodeling suggestive of impaired glucose-related inter-organ metabolic adaptation across skeletal muscle, liver, and kidney. Similar glycemic and sarcopenic features were observed when nephron loss was superimposed on aging, and reduced renal tubular *PCK1* expression was detected in older patients with CKD. Together, these findings support a framework in which cardiometabolic stress and aging impose overlapping constraints on glucose-related metabolic adaptation,^1^^,^^40^ while superimposed kidney dysfunction further compromises adaptive glucose homeostasis and skeletal muscle metabolic flexibility. This metabolic vulnerability may contribute to calorie-intake-independent wasting with sarcopenic and physical frailty features.

CKD is increasingly recognized as an accelerator of biological aging and a major risk factor for sarcopenia and frailty,^8^^,^^9^^,^^15^^,^^23^ yet nephron loss alone often does not reproduce overt sarcopenic phenotypes in experimental models.^25^^,^^26^ Clinical associations between CKD and sarcopenia are also observed largely in older individuals with multiple comorbidities,^10^^,^^11^^,^^22^^,^^41^ suggesting that aging-related vulnerability and cumulative systemic stress may be required for overt muscle loss to emerge. We therefore incorporated high-fat feeding and nitric oxide synthase inhibition into the nephrectomy model to reconstruct a cardiometabolic-stress component that is frequently embedded within aging-associated CKD, including lipid overload, vascular stress, hypertension-related stress, and HFpEF-like cardiometabolic dysfunction.^38^^,^^39^ This approach was not intended to model aging itself, but rather to isolate and intensify a metabolically relevant component of the aging-CKD milieu. We then examined aging-plus-nephron-loss mice to determine whether related nephron loss-associated wasting and impaired glycemic response were also observed within the broader context of physiological aging.

A notable feature of the HLN model is that it captured multiple core domains of sarcopenia, including reduced skeletal muscle mass, smaller myofibers, impaired grip strength, and reduced exercise endurance.^5^^,^^6^^,^^7^ This is important because experimental CKD-related muscle wasting models often reproduce selected components of sarcopenia rather than the combined loss of muscle mass, strength, and physical performance.^22^^,^^42^ The HLN phenotype was also not readily explained by mechanisms classically invoked in CKD-associated sarcopenia. Although CKD-related metabolic abnormalities are often discussed in terms of glucose intolerance and insulin resistance,^43^ HLN mice developed sarcopenic and physical frailty features despite lacking the glucose-intolerant and hyperinsulinemic phenotype observed in HL mice. Likewise, reduced intake, uremic burden, or systemic inflammation alone was insufficient to account for this phenotype, because caloric intake was preserved, renal dysfunction and TNFα elevation were comparable between N and HLN mice, whereas only HLN mice developed a sarcopenic phenotype across multiple domains. These findings point to a mismatch between caloric intake and tissue maintenance that is not explained by conventional mechanisms alone, and may reflect a clinically recognizable shift in CKD and aging from obesity- and glucose intolerance-dominant manifestations toward wasting and frailty.^16^^,^^44^^,^^45^

Skeletal muscle analyses provided insight into this mismatch between caloric intake and tissue maintenance. HLN muscle showed a downregulated transcriptomic signature including *Hk2* and *Mlxipl*, fasting-associated reductions in these glucose-related genes, reduced glycogen content, suppression of broader glucose-related gene programs, and accumulation of glycolytic metabolites. These changes occurred despite the absence of the glucose-intolerant phenotype observed in HL mice, suggesting that HLN mice develop suppressed skeletal muscle glucose-related metabolic programs in association with wasting rather than classical glucose intolerance alone.^46^^,^^47^ In addition, the lipid-utilization gene program induced by high-fat feeding in HL mice was not induced in HLN mice, and amino acid catabolic signatures were increased without sustained activation of canonical transcriptional atrophy programs. Thus, HLN skeletal muscle appears to enter a metabolically maladaptive wasting state characterized by impaired metabolic flexibility, rather than a simple degradation-driven atrophy program.

The liver and kidney findings further suggest that altered systemic glycemic adaptation accompanies this skeletal muscle phenotype. Hepatic metabolomic profiles of HL and HLN mice largely overlapped, indicating that cardiometabolic stress drove a major component of hepatic metabolic remodeling even in HLN mice, which did not develop overt hepatic steatosis. However, only HLN mice showed a blunted glycemic response to lactate-pyruvate challenge, suggesting that hepatic remodeling alone does not explain the impaired glycemic response. Importantly, the glycemic response during the LPTT reflects the net balance between glucose appearance and disappearance; thus, the blunted response in HLN mice could result from reduced gluconeogenic glucose production,^28^^,^^29^^,^^30^ accelerated glucose disposal, or both, which cannot be distinguished from the present data. Altered hepatic gluconeogenic gene expression in the cardiometabolic model, together with reduced hepatic and renal *Pck1* and *G6pc* expression with aging, is consistent with impaired gluconeogenic adaptation, although these findings do not establish reduced glucose production flux. In skeletal muscle, however, the metabolic profile did not support enhanced glucose utilization, as glucose-responsive and glycolytic pathways were reduced rather than activated. Glucose uptake and utilization in other tissues were not directly assessed, and tissue-specific redistribution or enhancement of glucose disposal therefore cannot be excluded. If present, increased glucose disposal outside skeletal muscle could further limit systemic glucose availability during metabolic challenge and thereby exacerbate substrate limitation in skeletal muscle, potentially contributing to the wasting phenotype. Thus, our findings support impaired adaptive glucose homeostasis while leaving the relative contributions of glucose production and disappearance unresolved.

The aging-plus-nephron-loss model was used to test whether the vulnerability identified in the HLN model was restricted to an artificial cardiometabolic stress setting or was also evident in physiological aging.^48^^,^^49^ Consistent with this rationale, nephron loss superimposed on aging recapitulated key sarcopenic, physical frailty-related, and glycemic features observed in HLN mice, including impaired glycemic response to lactate-pyruvate challenge and reduced hepatic and renal gluconeogenic gene expression. These findings suggest that the cardiometabolic-stress component isolated in the HLN model may represent one metabolically relevant dimension of aging-associated vulnerability, rather than a model-specific response. Consistently, older patients with CKD showed reduced renal tubular *PCK1* expression despite relatively preserved renal function and comparable glycemic indices, suggesting that age-associated impairment of renal glucose-related metabolic machinery may precede advanced loss of kidney function.

The cornstarch intervention suggests that key sarcopenic and physical frailty features of the HLN phenotype are modifiable. We selected cornstarch because slowly digestible cornstarch has been used to maintain glycemia during fasting intervals in hepatic glycogen storage diseases,^50^ providing a rationale for testing whether prolonged carbohydrate availability could buffer the fasting-associated glycemic vulnerability suggested by the HLN phenotype. Replacing dietary sucrose with slowly digestible cornstarch increased blood glucose levels after 8 h fasting without reversing kidney dysfunction, and improved treadmill endurance, gastrocnemius muscle mass, myofiber size, and serum albumin despite lower body weight and reduced adipose tissue mass. Cornstarch also partially restored selected glucose-related genes under fasting conditions, including *Hk2*. These findings suggest that carbohydrate-based nutritional modulation can improve muscle and frailty-related features in HLN mice, although they do not establish that these benefits specifically resulted from improved glycemic adaptation. The lack of improvement with carnitine supplementation further suggests that providing an alternative fatty acid-related substrate is insufficient to rescue the HLN muscle phenotype.

In conclusion, our findings suggest that kidney dysfunction can shift the metabolic consequences of cardiometabolic stress from glucose intolerance and weight gain toward calorie-intake-independent wasting with sarcopenic and physical frailty features. This transition was associated with impaired adaptive glucose homeostasis and skeletal muscle metabolic maladaptation, rather than reduced food intake, renal dysfunction alone, inflammation, or an HL-like glucose-intolerant phenotype. The partial rescue by cornstarch-based nutritional modulation further suggests that key features of this phenotype are modifiable. These findings support a framework in which a subset of CKD-associated sarcopenia and physical frailty reflects impaired inter-organ metabolic adaptation and provide a rationale for future studies examining hepatic-renal-muscle glucose fluxes in aging and CKD.

### Limitations of the study

Several limitations should be acknowledged. First, glucose flux was not quantified using isotope tracer approaches; therefore, the relative contributions of glucose production and disappearance to the blunted glycemic response, as well as the hepatic and renal contributions to glucose production, could not be precisely determined. Second, skeletal muscle glucose uptake, insulin-stimulated glucose disposal, glycolytic flux, and fatty acid oxidation flux were not directly measured. Thus, the muscle findings should be interpreted as evidence of altered metabolic programs rather than direct proof of impaired substrate utilization. Third, nephron loss affects multiple aspects of renal physiology beyond glucose metabolism, and renal gluconeogenesis was not manipulated in a cell-specific manner. Moreover, targeted perturbation of renal gluconeogenic enzymes such as G6PC or PCK1 may exert tubular cell-autonomous effects independently of systemic glycemic regulation, as kidney-specific G6pc deletion causes nephropathy and PCK1 contributes to tubular mitochondrial metabolic integrity. Future studies combining organ-specific glucose flux analysis with targeted renal metabolic perturbation will therefore be required to distinguish systemic from renal cell-autonomous metabolic effects.^35^^,^^36^^,^^37^^,^^51^ Fourth, the cornstarch intervention does not establish that its beneficial effects were specifically mediated by improved glycemic adaptation, because altered intestinal glucose delivery, postprandial nutrient handling, or gut-derived signals may also have contributed. Finally, the human biopsy analysis assessed renal PCK1 expression but did not directly measure renal gluconeogenic activity or systemic glucose metabolism.

## Resource availability

### Lead contact

Requests for further information and resources should be directed to and will be fulfilled by the lead contact, Yuka Sato (yuka.sato.yt@fujita-hu.ac.jp).

### Materials availability

This study did not generate new unique reagents.

### Data and code availability

• RNA-sequencing data generated in this study have been deposited in the Gene Expression Omnibus (GEO) under accession number GEO: GSE314852 and are publicly available at https://www.ncbi.nlm.nih.gov/geo/query/acc.cgi?acc=GSE314852.
• All other data supporting the findings of this study are available from the lead contact upon reasonable request.
• This paper does not report original code.
• Any additional information required to reanalyze the data reported in this paper is available from the lead contact upon request.

## Acknowledgments

This work was supported by a Grant-in-Aid for Scientific Research (10.13039/501100001691KAKENHI, Grant-in-Aid for Scientific Research C, grant no 26K11191) from the 10.13039/501100001691Japan Society for the Promotion of Science, a medical research grant from the 10.13039/100019868Chukyo Longevity Medical Research Promotion Foundation, and a research grant from the Nitto Foundation. We thank the Division for Medical Research Engineering, Nagoya University Graduate School of Medicine, for access to shared research facilities and for the technical assistance provided by their staff. We also thank Yuriko Sawa and Naoko Asano for their assistance with the preparation of histopathological specimens. Graphical abstract: created in BioRender. Sato, Y. (2026), https://BioRender.com/c6mvtds.

## Author contributions

M.K. and Y.S. conceived and designed the study. M.K., Y.S., and A.H. performed the experiments and analyzed the data. A.R. and T.I. contributed to the conceptualization of the study. K.M., N.K., and K.F. provided guidance on data interpretation and the direction of the study. Y.K., M.S., Y.N., K.S., C.O., and K.T. assisted with the experiments and data processing. M.T. and K.T. provided experimental resources. M.K. and Y.S. wrote the manuscript. T.K., K.K., and S.M. supervised the project. Y.S., N.T., and S.M. acquired funding. All authors reviewed and approved the final version of the manuscript for submission.

## Declaration of interests

The authors declare no competing interests.

## Declaration of generative AI and AI-assisted technologies in the manuscript preparation process

During the preparation of this manuscript, the authors used OpenAI ChatGPT to improve the English expression and enhance readability. The authors also used OpenAI Codex to organize and format the manuscript and key resources table. After using these tools/services, the authors reviewed and edited the content as required. They take full responsibility for the content of the published article.

## STAR★Methods

### Key resources table

**Table:** 

REAGENT or RESOURCE	SOURCE	IDENTIFIER
**Antibodies**		

PCK1 monoclonal antibody	Proteintech	Cat# 66862-1-Ig; RRID: AB_2882200

**Biological samples**		

Human kidney biopsy specimens from patients with nephrosclerosis	Nagoya University Hospital	N/A

**Chemicals, peptides, and recombinant proteins**		

Nω-Nitro-L-arginine methyl ester (L-NAME)	Cayman Chemical	Cat# 80210
Sodium L-lactate	FUJIFILM Wako Pure Chemical	Cat# 195–05965
Sodium pyruvate	FUJIFILM Wako Pure Chemical	Cat# 199–03062
Glucose	Nichi-Iko Pharmaceutical	N/A
High-fat diet (60% kcal from fat)	Research Diets, Inc.	Cat# D12492
Cornstarch-based diet	Research Diets, Inc.	Cat# D22062908
High-fat diet containing 0.5% L-carnitine	Research Diets, Inc.	Cat# D25030603

**Critical commercial assays**		

Mouse Glucagon ELISA Kit	FUJIFILM Wako Pure Chemical Corporation	Cat# 292–80001
Mouse Insulin ELISA Kit	Morinaga Institute of Biological Science, Inc.	Cat# M1101
RNeasy Mini Kit	QIAGEN	Cat# 74104
RNeasy Fibrous Tissue Mini Kit	QIAGEN	Cat# 74704
ReverTra Ace	Toyobo	Cat# FSQ-301
PrimeTime™ Gene Expression Master Mix	Integrated DNA Technologies (IDT)	Cat# 1055772
Glycogen Assay Kit	Abcam	Cat# ab65620
Pierce BCA Protein Assay Kit	Thermo Fisher Scientific	Cat#23225

**Deposited data**		

RNA-sequencing data	Gene Expression Omnibus (GEO)	GSE314852; https://www.ncbi.nlm.nih.gov/geo/query/acc.cgi?acc=GSE314852

**Experimental models: Organisms/strains**		

Mouse: C57BL/6J, male	Japan SLC	C57BL/6JmsSlc
Mouse: C57BL/6N, male	CLEA Japan	C57BL/6NJcl

**Oligonucleotides**		

Mouse Gapdh PrimeTime qPCR assay	Integrated DNA Technologies (IDT)	Mm.PT.39a.1
Mouse Actb PrimeTime qPCR assay	Integrated DNA Technologies (IDT)	Mm.PT.39a.22214843.g
Mouse G6pc PrimeTime qPCR assay	Integrated DNA Technologies (IDT)	Mm.PT.58.11964858
Mouse Pck1 PrimeTime qPCR assay	Integrated DNA Technologies (IDT)	Mm.PT.58.11992693
Mouse Mlxipl PrimeTime qPCR assay	Integrated DNA Technologies (IDT)	Mm.PT.56a.33592172
Mouse Hk2 PrimeTime qPCR assay	Integrated DNA Technologies (IDT)	Mm.PT.58.32698746
Mouse Mstn PrimeTime qPCR assay	Integrated DNA Technologies (IDT)	Mm.PT.58.13573446
Mouse Fbxo32 (Atrogin-1) PrimeTime qPCR assay	Integrated DNA Technologies (IDT)	Mm.PT.58.7025875
Mouse Trim63 (MuRF1) PrimeTime qPCR assay	Integrated DNA Technologies (IDT)	Mm.PT.58.32840172

**Software and algorithms**		

GraphPad Prism 10	GraphPad Software	RRID: SCR_002798
ImageJ	National Institutes of Health (NIH)	RRID: SCR_003070
MetaboAnalyst 5.0	McGill University	RRID: SCR_015539
iDEP2	South Dakota State University	RRID: SCR_015827
HALO Image Analysis Software	Indica Labs	RRID: SCR_018350
QuantStudio Design & Analysis Software	Applied Biosystems	RRID: SCR_018129

### Experimental model and study participant details

#### Animals

Male C57BL/6JmsSlc (Japan SLC) and C57BL/6NJcl (CLEA Japan) mice were used in this study. Male C57BL/6J mice were used for the aging and CKD models, whereas male C57BL/6N mice were used for the kidney dysfunction under cardiometabolic stress model and the associated intervention experiments.

Young mice were 8 weeks of age at the start of the experiments. For the aging experiments, aged male C57BL/6J mice were 74–82 weeks of age at the start of the study.

All mice were housed in a temperature- and humidity-controlled environment under a 12-h light/dark cycle with free access to water and standard chow unless otherwise specified. All animal experiments were performed in accordance with institutional guidelines for animal care and applicable regulations and were approved by the Animal Care and Use Committee of Nagoya University (approval number: M250252).

Only male mice were used in the animal experiments to maintain a consistent experimental framework across models. The HFD+L-NAME cardiometabolic stress model was originally established in male mice, and female mice have been reported to be less susceptible to this intervention. Consequently, potential sex-specific differences in the observed metabolic and muscle phenotypes were not evaluated, limiting the generalizability of these findings across sexes.

#### Human study participants

Kidney biopsy specimens from patients clinically diagnosed with nephrosclerosis at Nagoya University Hospital were retrospectively analyzed.

Among 210 patients diagnosed with nephrosclerosis between 2018 and 2025, patients with relatively preserved renal function, defined as serum creatinine ≤2.0 mg/dL, were considered for inclusion. Patients with diabetes, defined as HbA1c ≥ 7.0%, and specimens with insufficient cortical tissue were excluded.

Thirty patients were included in the final analysis, comprising 14 younger patients aged ≤50 years and 16 older patients aged ≥75 years. Both male and female participants were included. The study was not designed to evaluate sex-specific differences in renal PCK1 expression.

The study involving human kidney biopsy specimens were approved by the Institutional Review Board of the Nagoya University Graduate School of Medicine (Nagoya Kidney Disease Registry; 2010–1135), and informed consent was obtained from all participants.

### Method details

#### Cardiometabolic stress model

Eight-week-old male C57BL/6N mice were randomly assigned to four experimental groups: control (C), nephrectomy (N), high-fat diet combined with L-NAME (HL), and high-fat diet combined with L-NAME and nephrectomy (HLN).

The high-fat diet contained 60% kcal from fat (D12492; Research Diets, Inc., New Brunswick, NJ, USA), and L-NAME was administered in drinking water at 1 g/L.

The HFD + L-NAME intervention was used not to model aging itself, but to isolate and intensify a cardiometabolic-stress component frequently embedded within aging-associated CKD. The HLN model therefore represents kidney dysfunction superimposed on cardiometabolic stress.

Body weight and food intake were recorded weekly during the experimental period. Food was provided *ad libitum*. Mice were sacrificed 14 weeks after the initial nephrectomy for sample collection.

#### Aging and CKD models

Eight-week-old young and 74- to 82-week-old aged male C57BL/6J mice were used to examine the combined effects of aging and nephron loss.

Mice underwent either sham operation or subtotal nephrectomy (5/6 nephrectomy), generating four groups: young control (YC), young nephrectomized (YN), aged control (AC), and aged nephrectomized (AN).

Subtotal nephrectomy was performed in two steps: two-thirds resection of the left kidney followed by right nephrectomy 1 week later.^25^

Experiments were initiated with six mice per group. One mouse in the AN group died during the experimental period; therefore, five animals remained in this group at the end of the study. Body weight, skeletal muscle mass, and other terminal phenotypic outcomes were evaluated in all animals that survived until sacrifice.

For the lactate–pyruvate tolerance test, five animals were analyzed in each group to maintain equal sample sizes across groups. Accordingly, all five surviving animals in the AN group were included, whereas five animals were randomly selected from each of the YC, YN, and AC groups.

Body weight and food intake were recorded weekly. Mice were sacrificed 14 weeks after the initial surgery, and blood, liver, kidney, and skeletal muscle samples were collected for analysis.

#### Additional groups and dietary interventions

To dissect the contribution of individual components of cardiometabolic stress, additional nephrectomized groups receiving either L-NAME alone (LN) or high-fat diet alone (HN) were examined.

For nutritional intervention experiments, an additional HLN cohort was fed either a sucrose-based or cornstarch-based diet (D22062908; Research Diets, Inc.).

To examine whether altered fatty acid metabolism contributed to the HLN muscle phenotype, another HLN cohort received a high-fat diet containing 0.5% L-carnitine (D25030603; Research Diets, Inc.).

#### Grip strength test

Fore- and hindlimb grip strength was measured using a grip strength meter (Columbus Instruments, Columbus, OH, USA). Each mouse was tested three consecutive times, and the average value was recorded as maximal grip strength. Measurements were performed during the final week of the observation period at 14 weeks, and testing order and time of day were standardized across experimental groups.

#### Treadmill exercise test

Whole-body endurance and exercise tolerance were evaluated using a motorized treadmill (Muromachi Kikai Co., Ltd., Tokyo, Japan) set at a 0° incline.

Mice were initially run at 5 m/min for 4 min, followed by a gradual increase to 14 m/min over 2 min. Thereafter, speed was increased by 2 m/min every 2 min until exhaustion.

Exhaustion was defined as the point at which a mouse was unable to continue running and remained in contact with the rear stopper for >10 consecutive seconds. The test was performed on three consecutive days during the final week of the observation period, and the peak performance, defined as the best value among the three trials, was recorded. Exercise capacity was quantified as the product of body weight, running distance, and running speed.

#### Serum biochemical analysis

Blood samples were collected from the orbital venous plexus immediately before euthanasia. Blood urea nitrogen, serum creatinine, and albumin concentrations were measured by an external laboratory (Serkova Co., Ltd., Tokyo, Japan).

#### Histological analysis

Gastrocnemius muscles were excised after euthanasia and fixed in 10% neutral-buffered formalin, followed by graded ethanol dehydration and paraffin embedding.

Sections 5 μm in thickness were stained with hematoxylin and eosin. Images from multiple fields per section were acquired using a light microscope and analyzed using ImageJ software (National Institutes of Health, Bethesda, MD, USA).

For each sample, at least 90 muscle fibers were analyzed to determine the mean myofiber cross-sectional area. Histological analyses were performed blinded to group assignment.

#### Lactate–pyruvate tolerance test

To assess the systemic glycemic response to gluconeogenic substrate loading, a lactate–pyruvate tolerance test (LPTT) was performed.

After a 16-h fast, mice received an intraperitoneal injection of a mixture of lactate and pyruvate at a 10:1 ratio, with a total dose of 1 g/kg body weight.^52^

Blood glucose levels were measured at 0, 15, 30, 60, 90, and 120 min after injection using a glucometer. The glycemic response was evaluated as the area under the curve (AUC) of the change in blood glucose concentration (Δ-glucose) from baseline at 0 min.^53^

#### Intraperitoneal glucose tolerance test

The intraperitoneal glucose tolerance test (IPGTT) was performed after an 8-h fast. Mice received an intraperitoneal injection of glucose at 1 g/kg body weight.

Blood glucose levels were measured at 0, 15, 30, 60, 90, and 120 min after injection using a handheld glucometer(Johnson & Johnson K.K.).^52^ The AUC for blood glucose concentration was calculated to assess systemic glucose tolerance.

#### Gene expression analysis

Total RNA was extracted from liver and kidney tissues using the RNeasy Mini Kit (QIAGEN, Hilden, Germany). For gastrocnemius muscle, total RNA was extracted using the RNeasy Fibrous Tissue Mini Kit (QIAGEN).

RNA concentration and purity were assessed using a NanoDrop 2000 spectrophotometer. Complementary DNA was synthesized from 1 μg of total RNA using ReverTra Ace (Toyobo, Osaka, Japan).

Quantitative real-time PCR was performed using a QuantStudio Real-Time PCR System, PrimeTime Gene Expression Master Mix (Integrated DNA Technologies [IDT], Cat# 1055772), and gene-specific PrimeTime qPCR assays (IDT; assay IDs are listed in the key resources table).

Gene expression analyses were performed using four to five animals per group because of limitations in the number of samples that could be processed within each assay. Animals were selected without reference to gene expression results. To obtain samples representative of the phenotypic characteristics of each group, animals whose body weights at sacrifice were closest to the group median were selected.

Relative gene expression levels were calculated using the ΔΔCt method and normalized to *Actb* expression for liver and kidney samples and *Gapdh* expression for skeletal muscle samples. Technical duplicate measurements were averaged for each biological sample and were not treated as independent biological replicates.

For kidney gene expression analysis, samples from animals subjected to 5/6 nephrectomy were excluded because tissue loss and surgery-associated structural alterations precluded consistent and representative tissue sampling.

#### Hormone measurements

Blood samples were collected from the orbital venous plexus under anesthesia at the time of sacrifice.

Serum glucagon levels were measured using a Mouse Glucagon ELISA Kit (FUJIFILM Wako Pure Chemical Corporation), and plasma insulin levels were measured using a Mouse Insulin ELISA Kit (Morinaga Institute of Biological Science, Inc.).

All assays were performed according to the manufacturers’ protocols, and each biological sample was analyzed as a single measurement.

#### Muscle glycogen analysis

Skeletal muscle glycogen content was measured using a Glycogen Assay Kit (Abcam, Cambridge, UK; ab65620) according to the manufacturer’s protocol.

Frozen skeletal muscle samples were homogenized in the supplied assay buffer and centrifuged to remove insoluble debris. Supernatants were incubated with glucoamylase to hydrolyze glycogen into glucose. The generated glucose was detected using a colorimetric reaction, and absorbance was measured at 570 nm.

Glycogen concentrations were calculated using a glycogen standard curve. Protein concentrations in the same lysates were determined using the Pierce BCA Protein Assay Kit (Thermo Fisher Scientific, Cat# 23225). Glycogen levels were normalized to protein content and expressed as μg glycogen per mg protein.

#### Metabolomic analysis

Untargeted metabolomic profiling of serum, gastrocnemius muscle, and liver samples was performed using capillary electrophoresis–time-of-flight mass spectrometry (CE-TOFMS).^54^

Metabolomic data were processed and statistically analyzed using MetaboAnalyst version 5.0. Metabolite levels were normalized using *Z* score transformation before visualization. Heat maps were generated using GraphPad Prism 10.

#### RNA sequencing and bioinformatics analysis

RNA concentration and purity were measured using a NanoDrop spectrophotometer, and RNA integrity was assessed using an Agilent 2100 Bioanalyzer.

Library preparation and sequencing were performed by Takara Bio Inc. mRNA was processed using a standard mRNA-seq library preparation protocol, and sequencing was performed on an Illumina platform with paired-end 150-bp reads.

FASTQ files were analyzed using the integrated iDEP2 pipeline for alignment, normalization, differential expression, and pathway enrichment analyses.

Genes with an FDR <0.1 and |fold change| > 1.5 were considered significantly differentially expressed. Gene Ontology and pathway enrichment analyses were performed using iDEP2. Heat maps were generated using GraphPad Prism 10.

The RNA-sequencing dataset generated in this study has been deposited in GEO under accession number GSE314852.

#### Renal PCK1 immunohistochemistry

Kidney biopsy specimens were subjected to immunohistochemical staining using a PCK1 monoclonal antibody (Proteintech, Cat# 66862-1-Ig). PCK1-positive tubular areas were quantified using HALO image analysis software (Indica Labs, Albuquerque, NM, USA).

### Quantification and statistical analysis

All statistical analyses were performed using GraphPad Prism version 10.0. Data are reported as mean ± SD unless otherwise indicated. For animal experiments, *n* represents the number of individual animals and therefore represents biological replicates. For the human kidney biopsy analysis, *n* represents the number of individual participants. Technical replicates were not treated as independent biological replicates.

Two-group comparisons were performed using unpaired two-tailed Student’s *t* tests or Mann–Whitney U tests, as appropriate. Comparisons among three or more groups were performed using one-way analysis of variance (ANOVA) followed by Tukey’s multiple comparisons test. Categorical variables were compared using the chi-square test. Pearson’s correlation was used to calculate correlation coefficients where appropriate.

Statistical significance was defined as *p* < 0.05. Where statistical significance is indicated by asterisks, *p* < 0.05 is denoted by ∗, *p* < 0.01 by ∗∗, *p* < 0.001 by ∗∗∗, and *p* < 0.0001 by ∗∗∗∗. The statistical test used for each experiment, exact sample sizes, and definitions of the data presented are provided in the corresponding figure legends. Unless otherwise indicated, error bars represent SD.
