# Supplementary material for: Kidney dysfunction under cardiometabolic stress promotes calorie-intake-independent wasting and frailty-like muscle phenotypes
Source: iScience. 2026 Oct 30;29(11):118058. doi: 10.1016/j.isci.2026.118058 (PMC13742467; doi:10.1016/j.isci.2026.118058)
Supplement: Supplementary file 1 [file mmc1.pdf]

iScience, Volume *29*

Supplemental information

Kidney dysfunction under cardiometabolic stress promotes calorie-intake-independent wasting and frailty-like muscle phenotypes

Mitsuharu Kojima, Yuka Sato, Akiyoshi Hirayama, Akihiro Ryuge, Takahiro Imaizumi, Kayaho Maeda, Noritoshi Kato, Yohei Kozaki, Masahiro Sugimura, Yuhei Noda, Katsuaki Shibata, Chikao Onogi, Kota Tanabe, Mikito Takefuji, Kenya Terabe, Kazuhiro Furuhashi, Tomoki Kosugi, Naotake Tsuboi, Kenji Kadomatsu, and Shoichi Maruyama

Supplementary Figure S1. Cardiometabolic and body-composition features of the HLN model

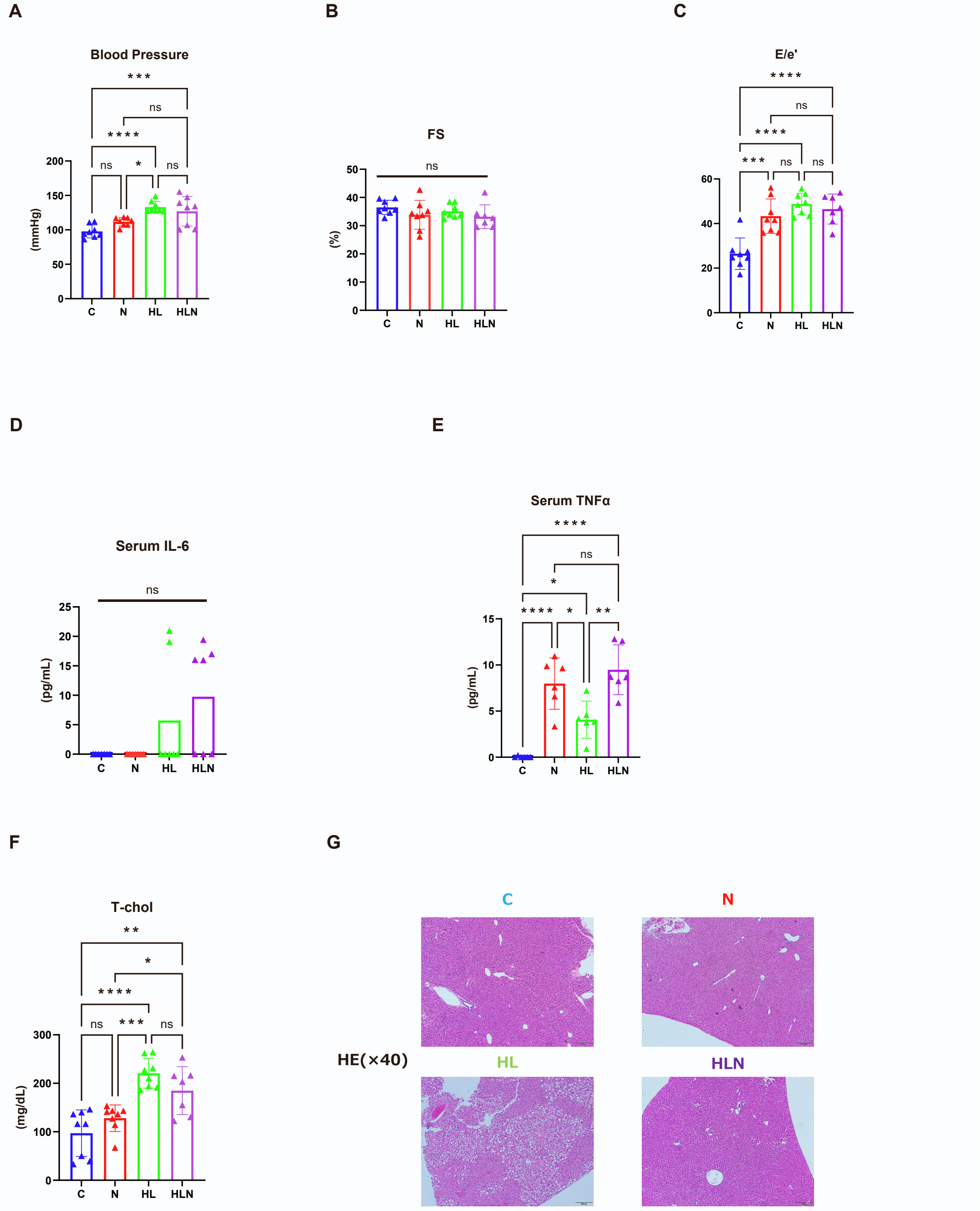

Supplementary Figure S1. Cardiometabolic and body-composition features of the HLN model

C, control; N, nephrectomy alone; HL, high-fat diet plus L-NAME; HLN, nephrectomy plus high-fat diet and L-NAME. L-NAME, Nω-nitro-L-arginine methyl ester.

(A) Systolic blood pressure (C, n = 8; N, n = 8; HL, n = 8; HLN, n = 8).

(B) Fractional shortening (FS; C, n = 8; N, n = 8; HL, n = 8; HLN, n = 7).

(C) Ratio of early transmitral inflow velocity to early diastolic mitral annular velocity (E/e′; C, n = 8; N, n = 8; HL, n = 8; HLN, n = 7).

(D) Serum interleukin-6 (IL-6) concentration (C, n = 6; N, n = 6; HL, n = 6; HLN, n = 7).

(E) Serum tumor necrosis factor α (TNFα) concentration (C, n = 6; N, n = 6; HL, n = 6; HLN, n = 6).

(F) Serum total cholesterol (T-chol) concentration (C, n = 8; N, n = 8; HL, n = 8; HLN, n = 7).

(G) Representative hematoxylin and eosin (H&E)-stained liver sections. Scale bars, 200 μm.

Data are presented as mean ± SD (standard deviation). Statistical comparisons were performed using one-way analysis of variance (ANOVA) followed by Tukey's multiple-comparison test. *p < 0.05, **p < 0.01, ***p < 0.001, and ****p < 0.0001; ns, not significant.

Supplementary Figure S2. Contribution of high-fat feeding and L-NAME treatment to the HLN phenotype in nephrectomized mice

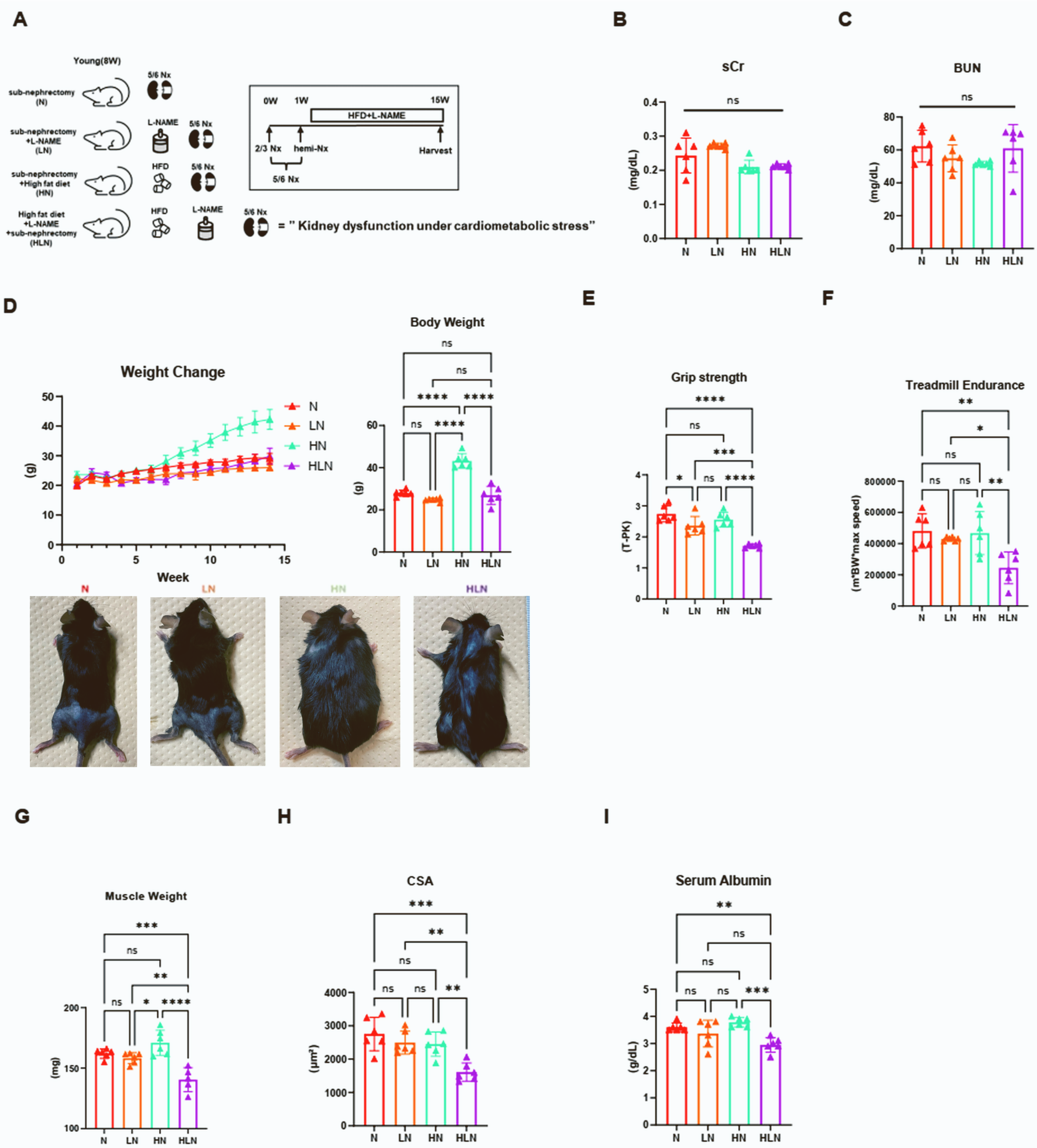

Supplementary Figure S2. Contribution of high-fat feeding and L-NAME treatment to the HLN phenotype in nephrectomized mice

(A) Experimental groups and timeline. Mice underwent two-stage 5/6 nephrectomy (Nx), with 2/3 nephrectomy at week 0 and contralateral heminephrectomy at week 1; tissues were collected at week 15. N, nephrectomy alone; LN, nephrectomy plus L-NAME; HN, nephrectomy plus high-fat diet (HFD); HLN, nephrectomy plus HFD and L-NAME. L-NAME, Nω-nitro-L-arginine methyl ester.

(B) Serum creatinine (sCr) concentration. (C) Blood urea nitrogen (BUN) concentration.

(D) Body weight over time (left), body weight at tissue collection (right), and representative photographs of mice (bottom).

(E) Grip strength. (F) Treadmill endurance.

(G) Gastrocnemius muscle weight. (H) Myofiber cross-sectional area (CSA). (I) Serum albumin concentration.

For the quantitative measurements in (B–I), N, $n = 6$; LN, $n = 6$; HN, $n = 6$; HLN, $n = 6$. Data are presented as mean ± SD (standard deviation). Single-time-point measurements in (B–I) were compared using one-way analysis of variance (ANOVA) followed by Tukey's multiple-comparison test. *$p < 0.05$, **$p < 0.01$, ***$p < 0.001$, and ****$p < 0.0001$; ns, not significant.

Supplementary Figure S3. Additional skeletal muscle transcriptomic and metabolomic analyses in HLN mice

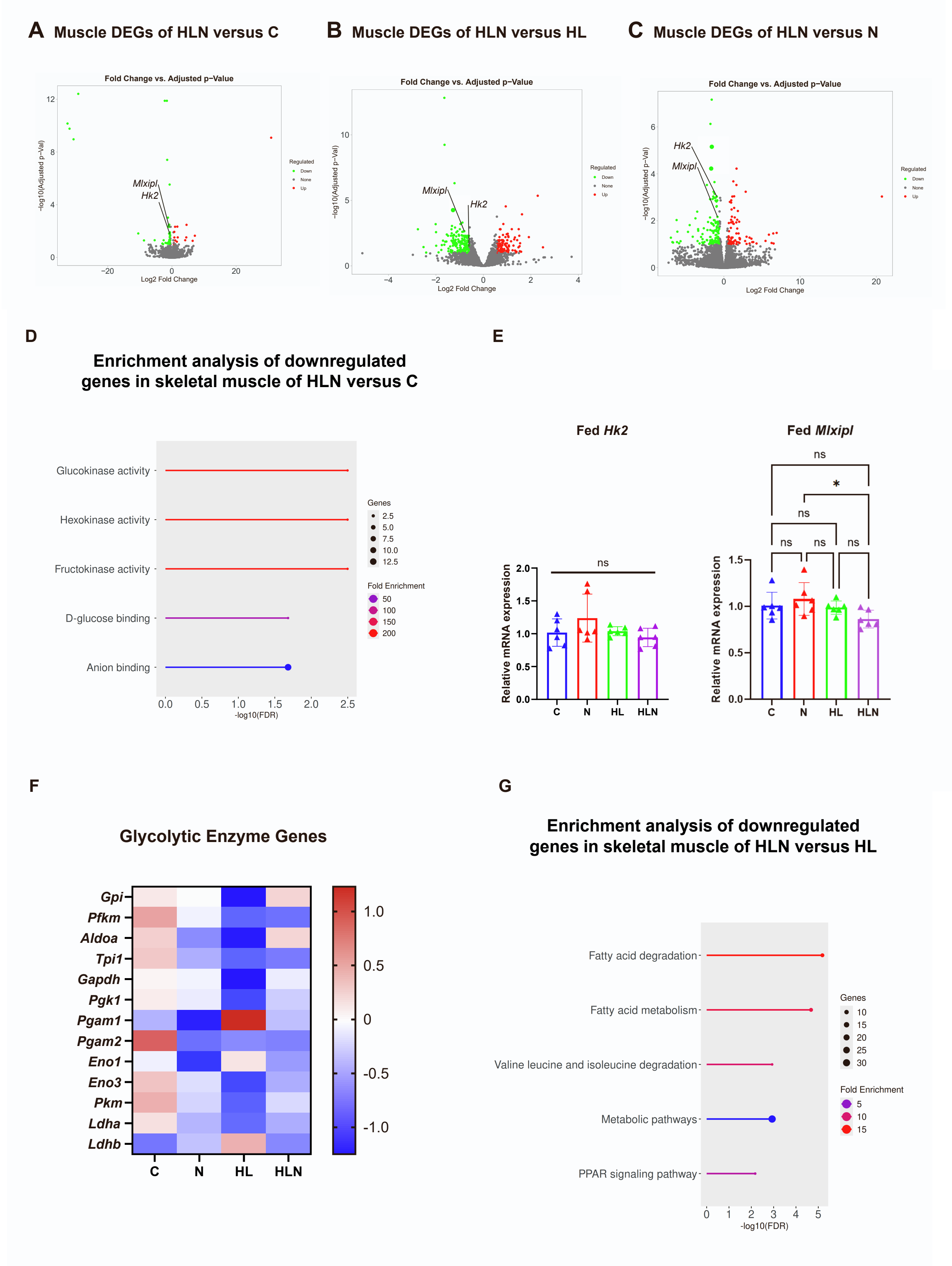

Supplementary Figure S3. Additional skeletal muscle transcriptomic and metabolomic analyses in HLN mice

C, control; N, nephrectomy alone; HL, high-fat diet plus L-NAME; HLN, nephrectomy plus high-fat diet and L-NAME. L-NAME, Nω-nitro-L-arginine methyl ester.

(A–C) Volcano plots of skeletal muscle gene expression in HLN versus C (A), HL (B), and N (C) mice (C, n = 3; N, n = 3; HL, n = 3; HLN, n = 3). The horizontal axes show log2 fold change, and the vertical axes show −log10(adjusted p value). Green, downregulated genes; red, upregulated genes; gray, genes not classified as differentially expressed. *Hk2* and *Mlxipl* are labeled.

(D) Gene Ontology (GO) molecular function enrichment analysis of genes downregulated in HLN versus C skeletal muscle (C, n = 3; HLN, n = 3). The horizontal axis shows −log10(false discovery rate [FDR]); point size indicates gene count, and color indicates fold enrichment.

(E) Relative skeletal muscle *Hk2* (hexokinase 2) and *Mlxipl* (MLX interacting protein like) mRNA expression under fed conditions (C, n = 5; N, n = 5; HL, n = 5; HLN, n = 5). Data are presented as mean ± SD (standard deviation). Groups were compared using one-way analysis of variance (ANOVA) followed by Tukey's multiple-comparison test. *$p < 0.05$; ns, not significant.

(F) Heatmap of glycolytic gene expression in skeletal muscle (C, n = 3; N, n = 3; HL, n = 3; HLN, n = 3). Columns represent the indicated groups; red and blue denote higher and lower values, respectively.

(G) Kyoto Encyclopedia of Genes and Genomes (KEGG) pathway enrichment analysis of genes downregulated in HLN versus HL skeletal muscle (HL, n = 3; HLN, n = 3). The horizontal axis shows −log10(FDR); point size indicates gene count, and color indicates fold enrichment. PPAR, peroxisome proliferator-activated receptor.

Supplementary Figure S4. Additional hepatic metabolomic analyses in the HLN model

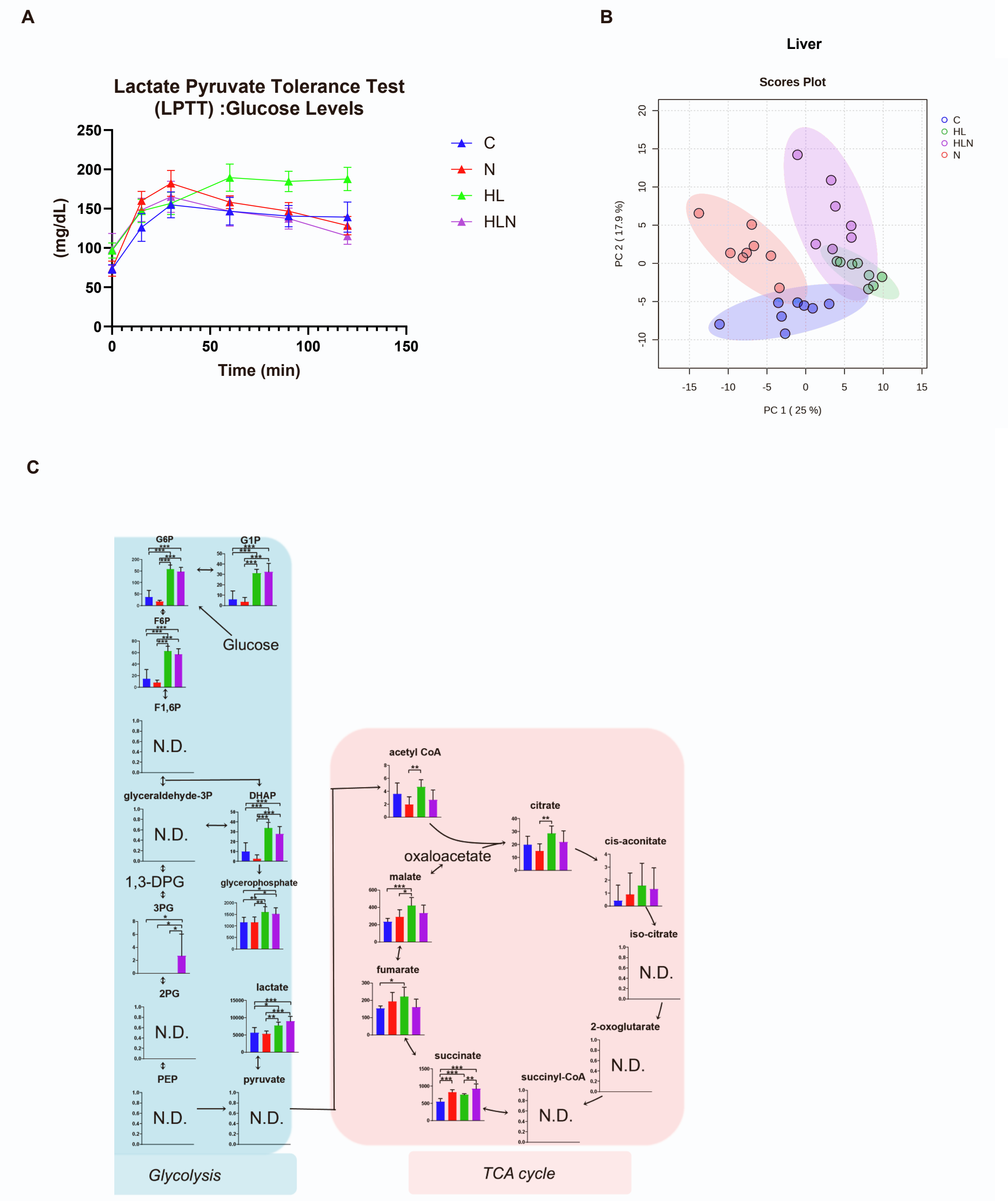

Supplementary Figure S4. Additional hepatic metabolomic analyses in the HLN model

C, control; N, nephrectomy alone; HL, high-fat diet plus L-NAME; HLN, nephrectomy plus high-fat diet and L-NAME. L-NAME, Nω-nitro-L-arginine methyl ester.

(A) Blood glucose concentrations during the lactate–pyruvate tolerance test (LPTT; C, n = 8; N, n = 8; HL, n = 7; HLN, n = 8). Error bars indicate SD (standard deviation).

(B) Principal component analysis (PCA) scores plot of hepatic metabolomic profiles (C, n = 8; N, n = 8; HL, n = 8; HLN, n = 7). PC1 and PC2 explain 25.0% and 17.9% of the variance, respectively.

(C) Hepatic metabolite profiles mapped onto glycolysis and the tricarboxylic acid (TCA) cycle (C, n = 8; N, n = 8; HL, n = 8; HLN, n = 7). Bars are ordered C, N, HL, and HLN from left to right and are colored blue, red, green, and purple, respectively. N.D., no data.

G6P, glucose 6-phosphate; G1P, glucose 1-phosphate; F6P, fructose 6-phosphate; F1,6P, fructose 1,6-bisphosphate; glyceraldehyde-3P, glyceraldehyde 3-phosphate; DHAP, dihydroxyacetone phosphate; 1,3-DPG, 1,3-bisphosphoglycerate; 3PG, 3-phosphoglycerate; 2PG, 2-phosphoglycerate; PEP, phosphoenolpyruvate; CoA, coenzyme A.

In (C), *$p < 0.05$, **$p < 0.01$, ***$p < 0.001$, and ****$p < 0.0001$.

Supplementary Figure S5. Additional data from the aging and nephron loss model

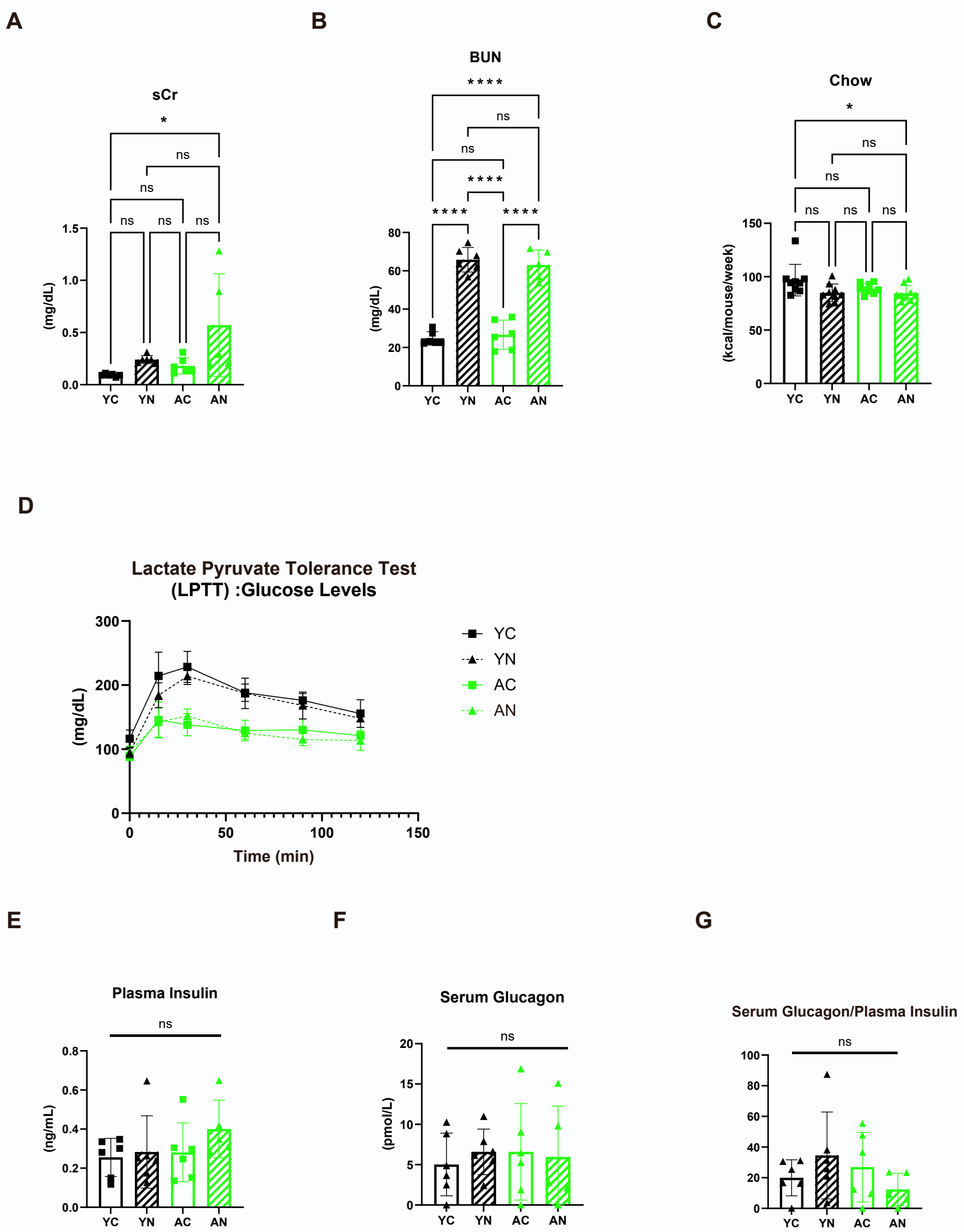

Supplementary Figure S5. Additional data from the aging and nephron loss model

YC, young control; YN, young nephrectomized; AC, aged control; AN, aged nephrectomized.

(A) Serum creatinine (sCr) concentration. (B) Blood urea nitrogen (BUN) concentration. For (A) and (B), YC, $n = 6$; YN, $n = 6$; AC, $n = 6$; AN, $n = 5$.

(C) Energy intake (kcal per mouse per week; YC, $n = 9$; YN, $n = 9$; AC, $n = 9$; AN, $n = 9$).

(D) Blood glucose concentrations during the lactate–pyruvate tolerance test (LPTT; YC, $n = 6$; YN, $n = 6$; AC, $n = 6$; AN, $n = 5$).

(E) Fasting plasma insulin concentration. (F) Fasting serum glucagon concentration. (G) Ratio of serum glucagon to plasma insulin. For (E–G), YC, $n = 6$; YN, $n = 6$; AC, $n = 6$; AN, $n = 5$.

Data are presented as mean ± SD (standard deviation). Groups in (A–C) and (E–G) were compared using one-way analysis of variance (ANOVA) followed by Tukey's multiple-comparison test. *$p < 0.05$ and ****$p < 0.0001$; ns, not significant.

Supplementary Figure S6. Additional effects of cornstarch feeding in HLN mice

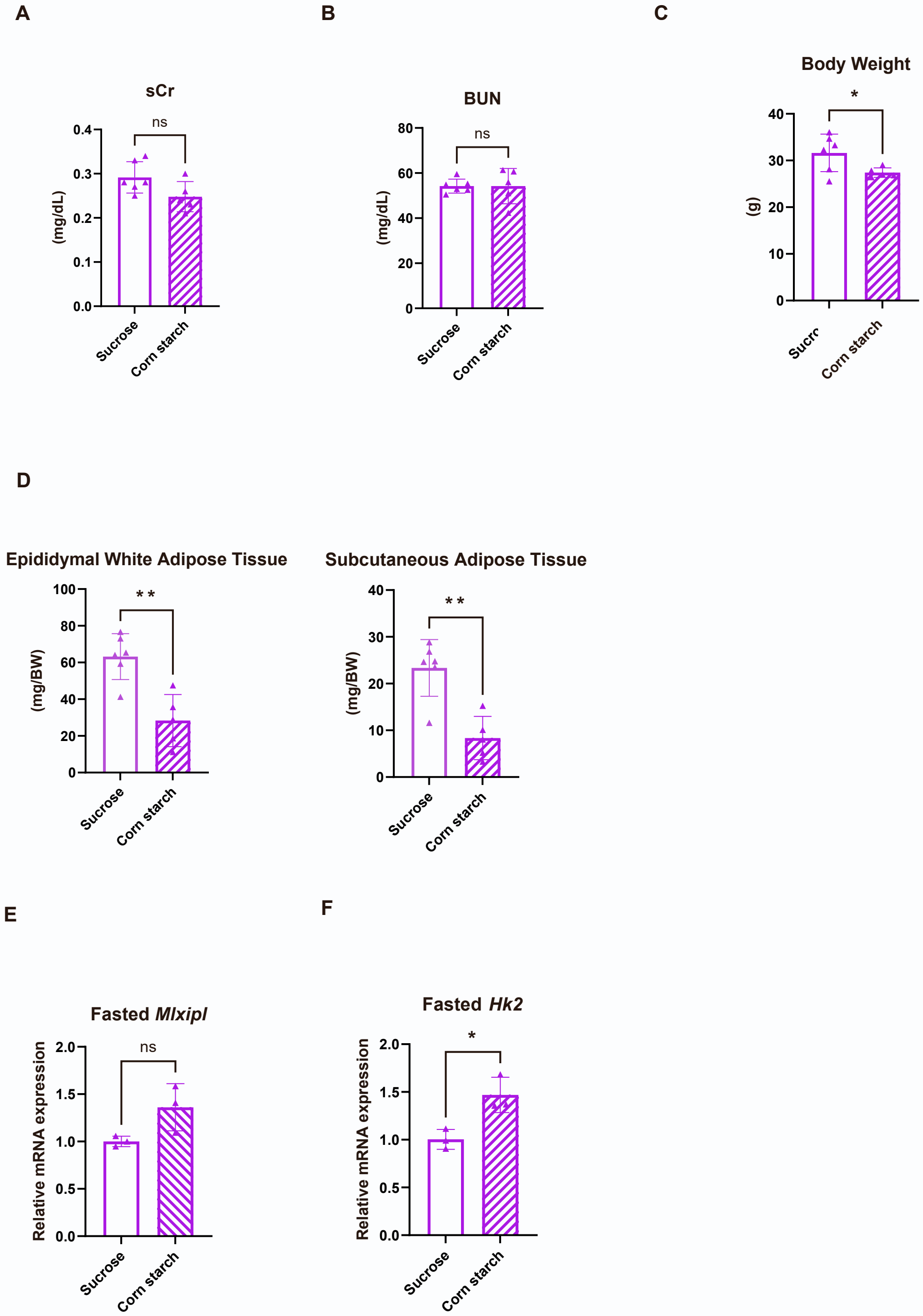

Supplementary Figure S6. Additional effects of cornstarch feeding in HLN mice

HLN mice (nephrectomy plus high-fat diet and L-NAME treatment) were fed sucrose- or cornstarch-based diets. L-NAME, Nω-nitro-L-arginine methyl ester.

(A) Serum creatinine (sCr) concentration. (B) Blood urea nitrogen (BUN) concentration. (C) Body weight at tissue collection.

(D) Epididymal white adipose tissue weight (left) and subcutaneous adipose tissue weight (right), normalized to body weight (BW). For (A–D), Sucrose, $n = 6$; Corn starch, $n = 6$.

(E) Relative skeletal muscle *Mlxipl* (MLX interacting protein like) mRNA expression under fasting conditions. (F) Relative skeletal muscle *Hk2* (hexokinase 2) mRNA expression under fasting conditions. For each panel, Sucrose, $n = 3$; Corn starch, $n = 3$.

Data are presented as mean ± SD (standard deviation). Diet groups were compared using unpaired, two-tailed Student's t tests. *$p < 0.05$ and **$p < 0.01$; ns, not significant.

Supplementary Figure S7. Carnitine supplementation does not ameliorate frailty-like muscle phenotypes in CKD-associated wasting models

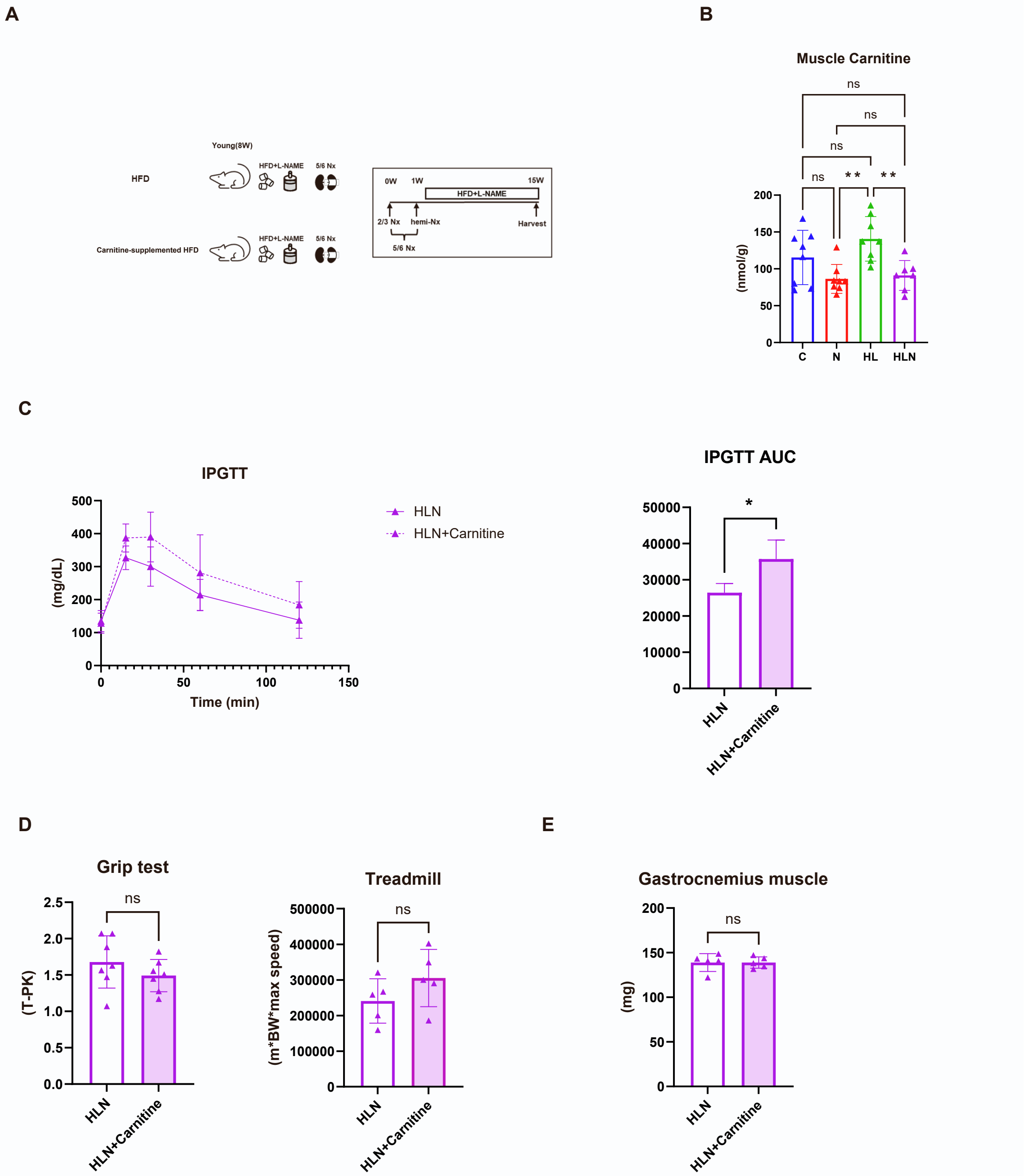

Supplementary Figure S7. Carnitine supplementation does not ameliorate frailty-like muscle phenotypes in CKD-associated wasting models

(A) Experimental design for dietary carnitine supplementation in HLN mice. HFD, high-fat diet; Nx, nephrectomy; L-NAME, Nω-nitro-L-arginine methyl ester.

(B) Skeletal muscle carnitine concentration in C (control), N (nephrectomy alone), HL (HFD plus L-NAME), and HLN (nephrectomy plus HFD and L-NAME) mice (C, n = 8; N, n = 8; HL, n = 8; HLN, n = 7).

(C) Blood glucose concentrations during the intraperitoneal glucose tolerance test (IPGTT; left) and the corresponding area under the curve (AUC; right) in HLN mice with or without carnitine supplementation (HLN, n = 6; HLN+Carnitine, n = 6).

(D) Grip strength (left) and treadmill endurance (right) in HLN mice with or without carnitine supplementation (HLN, n = 6; HLN+Carnitine, n = 6).

(E) Gastrocnemius muscle weight in HLN mice with or without carnitine supplementation (HLN, n = 6; HLN+Carnitine, n = 6).

Data are presented as mean ± SD (standard deviation). Groups in (B) were compared using one-way analysis of variance (ANOVA) followed by Tukey’s multiple-comparison test. The two groups in (C, AUC), (D), and (E) were compared using unpaired, two-tailed Student’s t tests. *$p < 0.05$ and **$p < 0.01$; ns, not significant.
